# Supplementary material for: A mathematical model of COVID-19 with multiple variants of the virus under optimal control in Ghana
Source: PLoS One. 2024 Jul 2;19(7):e0303791. doi: 10.1371/journal.pone.0303791 (PMC11218976; doi:10.1371/journal.pone.0303791)
Supplement: S1 Appendix — (PDF) [file pone.0303791.s001.pdf]

# Supporting information

## 0.1 Computation of Basic Reproduction Number ($R_0$)

The new generation matrix was used to derive the basic reproduction number. $\mathcal{F}$ is the rate of appearance of new infections and $\mathcal{V}$ is the rate of transfer of individuals who are not included in the secondary infections.

We considered the model equation (2 in the paper) and regrouped them into the disease class and non-disease class. The disease class is the exposed and infected compartments and the non-disease class (the other compartments) are healthy.

$$\mathcal{F} = \begin{pmatrix} \beta_1 I_1 S \\ \beta_2 I_2 S \\ 0 \\ 0 \end{pmatrix} \tag{1}$$

$$\mathcal{V} = \begin{pmatrix} (\mu+\sigma_1)E_1 \\ (\mu+\sigma_2)E_2 \\ -\sigma_1 E_1 + (\delta+\mu+\alpha_1+\gamma_1)I_1 \\ -\sigma_2 E_2 + (\delta+\mu+\alpha_2+\gamma_2)I_2 \end{pmatrix} \tag{2}$$

We compute F and V matrix by differentiating partially with respect to the original dependent variables. Let $x = E_1, E_2, I_1, I_2, Q = x_1, x_2, x_3, x_4, x_5$

$$F = \begin{pmatrix} \frac{\partial F_1}{\partial x_1} & \frac{\partial F_1}{\partial x_2} & \frac{\partial F_1}{\partial x_3} & \frac{\partial F_1}{\partial x_4} \\ \frac{\partial F_2}{\partial x_1} & \frac{\partial F_2}{\partial x_2} & \frac{\partial F_2}{\partial x_3} & \frac{\partial F_2}{\partial x_4} \\ \frac{\partial F_3}{\partial x_1} & \frac{\partial F_3}{\partial x_2} & \frac{\partial F_3}{\partial x_3} & \frac{\partial F_3}{\partial x_4} \\ \frac{\partial F_4}{\partial x_1} & \frac{\partial F_4}{\partial x_2} & \frac{\partial F_4}{\partial x_3} & \frac{\partial F_4}{\partial x_4} \end{pmatrix} = \begin{pmatrix} 0 & 0 & \beta_1 S & 0 \\ 0 & 0 & 0 & \beta_2 S \\ 0 & 0 & 0 & 0 \\ 0 & 0 & 0 & 0 \end{pmatrix} \tag{3}$$

At DFE $(S^*, I^*) = (1, 0)$

$$F = \begin{pmatrix} 0 & 0 & \beta_1 & 0 \\ 0 & 0 & 0 & \beta_2 \\ 0 & 0 & 0 & 0 \\ 0 & 0 & 0 & 0 \end{pmatrix} \tag{4}$$

$$V = \begin{pmatrix} \frac{\partial V_1}{\partial x_1} & \frac{\partial V_1}{\partial x_2} & \frac{\partial V_1}{\partial x_3} & \frac{\partial V_1}{\partial x_4} \\ \frac{\partial V_2}{\partial x_1} & \frac{\partial V_2}{\partial x_2} & \frac{\partial V_2}{\partial x_3} & \frac{\partial V_2}{\partial x_4} \\ \frac{\partial V_3}{\partial x_1} & \frac{\partial V_3}{\partial x_2} & \frac{\partial V_3}{\partial x_3} & \frac{\partial V_3}{\partial x_4} \\ \frac{\partial V_4}{\partial x_1} & \frac{\partial V_4}{\partial x_2} & \frac{\partial V_4}{\partial x_3} & \frac{\partial V_4}{\partial x_4} \end{pmatrix} = \begin{pmatrix} a & 0 & 0 & 0 \\ 0 & b & 0 & 0 \\ -\sigma_1 & 0 & c & 0 \\ 0 & -\sigma_2 & 0 & d \end{pmatrix} \tag{5}$$

The basic reproduction number is given by $R_0 = \rho(FV^{-1})$, where $\rho$ is the spectral radius. We compute inverse matrix of V ($V^{-1}$)

$$V^{-1} = \begin{pmatrix} \frac{1}{a} & 0 & 0 & 0 \\ 0 & \frac{1}{b} & 0 & 0 \\ \frac{\sigma_1}{ac} & 0 & \frac{1}{c} & 0 \\ 0 & \frac{\sigma_2}{bd} & 0 & \frac{1}{d} \end{pmatrix} \tag{6}$$

We further compute $FV^{-1}$

$$FV^{-1} = \begin{pmatrix} \frac{\beta_1\sigma_1}{ac} & 0 & \frac{\beta_1}{c} & 0 \\ 0 & \frac{\beta_2\sigma_2}{bd} & 0 & \frac{\beta_2}{d} \\ 0 & 0 & 0 & 0 \\ 0 & 0 & 0 & 0 \end{pmatrix} \tag{7}$$

To solve for $R_0 = \rho(FV^{-1})$, we find the eigenvalues of $FV^{-1}$. Therefore, the basic reproduction number is given by

$$R_0 = \max(R_0^1, R_0^2) \tag{8}$$

with

$$R_0^1 = \frac{\beta_1\sigma_1}{ac} \tag{9}$$

and

$$R_0^2 = \frac{\beta_2\sigma_2}{bd} \tag{10}$$

Recall that $a = \mu + \sigma_1,\; b = \mu + \sigma_2,\;\; c = \delta + \mu + \alpha_1 + \gamma_1,\; d = \delta + \mu + \alpha_2 + \gamma_2$ and $R_0^1$ is strain 1 reproduction number and $R_0^2$ is strain 2 reproduction number.

## 0.2 Proof of Existence of the Equilibrium Points

We show the detailed proof of theorem 3.4.

We consider the equilibrium points at the disease-free equilibrium ($\varepsilon_0$), strain 1 endemic equilibrium ($\varepsilon_1$), and strain 2 endemic equilibrium ($\varepsilon_2$). We solve equation (11 in the paper) to get the equilibria. The equilibria equation (11 in the paper) is restated as:

$$\frac{dS}{dt} = \Lambda - \beta_1 I_1 S - \beta_2 I_2 S - \mu S + \upsilon R \tag{11}$$

$$\frac{dE_1}{dt} = \beta_1 I_1 S - (\mu + \sigma_1)E_1 \tag{12}$$

$$\frac{dE_2}{dt} = \beta_2 I_2 S - (\mu + \sigma_2)E_2 \tag{13}$$

$$\frac{dI_1}{dt} = \sigma_1 E_1 - (\delta + \mu + \alpha_1 + \gamma_1)I_1 \tag{14}$$

$$\frac{dI_2}{dt} = \sigma_2 E_2 - (\delta + \mu + \alpha_2 + \gamma_2)I_2 \tag{15}$$

$$\frac{dQ}{dt} = \alpha_1 I_1 + \alpha_2 I_2 - (\mu + \theta)Q \tag{16}$$

At the disease free equilibrium ($\varepsilon_0$), there is no disease therefore $I_1 = 0$, $I_2 = 0$, and $R = 0$. Equations (11) - (16) were analyzed considering $I_1 = 0$, $I_2 = 0$, and $R = 0$. To verify the disease-free equilibrium (DFE), we set the equations to zero. From equation (11), we set $\frac{dS}{dt} = 0$. Then we have $\Lambda - \beta_1 I_1 S - \beta_2 I_2 S - \mu S + \upsilon R = 0$. Since $I_1 = 0, I_2 = 0, R = 0$, we have $\Lambda - \mu S = 0$ and then $\Lambda = \mu S$. Hence, $S = \frac{\Lambda}{\mu}$.

From equation (12), we set $\frac{dE_1}{dt} = 0$. Then we have $\beta_1 I_1 S - (\mu + \sigma_1)E_1 = 0$. If we consider $I_1 = 0$, and $I_2 = 0$. Therefore we get $0 = (\mu + \sigma_1)E_1$. Therefore we obtain $E_1 = 0$.

From equation (13), we set $\frac{dE_2}{dt} = 0$. Then we have $\beta_2 I_2 S - (\mu + \sigma_2)E_2 = 0$. If we consider $I_1 = 0, I_2 = 0$, and $R = 0$, we have $0 = (\mu + \sigma_2)E_2$. Therefore, we obtain $E_2 = 0$.

From equation (14), we set $\frac{dI_1}{dt} = 0$. Then we have $\sigma_1 E_1 - (\delta + \mu + \alpha_1 + \gamma_1)I_1 = 0$. And then $\frac{\sigma_1 E_1}{\delta + \mu + \alpha_1 + \gamma_1} = I_1$. We recall that $E_1 = 0$, therefore $I_1 = 0$.

From equation (15), we set $\frac{dI_2}{dt} = 0$. Then we have $\sigma_2 E_2 - (\delta + \mu + \alpha_2 + \gamma_2)I_2 = 0$. Then we obtain $\frac{\sigma_2 E_2}{\delta + \mu + \alpha_1 + \gamma_2} = I_2$. We recall that $E_2 = 0$, therefore we have $I_2 = 0$.

From equation (16), we set $\frac{dQ}{dt} = 0$. Then we have $\alpha_1 I_1 + \alpha_2 I_2 - (\mu + \theta)Q = 0$. If we consider $I_1 = 0$, and $I_2 = 0$, then $-(\mu + \theta)Q = 0$. Therefore, $Q = 0$.

At the disease free equilibrium, $\varepsilon_0 = \left(\frac{\Lambda}{\mu}, 0, 0, 0, 0, 0\right)$.

The endemic equilibrium ($\varepsilon_1$) for strain 1 was computed. At $\varepsilon_1$, $I_1 \neq 0$ and $I_2 = 0$. From equation (11), we set $\frac{dS}{dt} = 0$. Then we have $\Lambda - \beta_1 I_1 S - \beta_2 I_2 S - \mu S + \upsilon R = 0$. If we consider $I_1 \neq 0, I_2 = 0$, and $R = 0$, we have $\Lambda - \beta_1 I_1 S - \mu S = 0$. Then, $\Lambda - \mu S = \beta_1 I_1 S$. We further consider $\Lambda = \beta_1 I_1 S + \mu S$, then $\Lambda = (\beta_1 I_1 + \mu)S$. Therefore, $S = S_1^* = \frac{\Lambda}{\beta_1 I_1 + \mu}$

From equation (12), we set $\frac{dE_1}{dt} = 0$. Then we have $\beta_1 I_1 S - (\mu + \sigma_1)E_1 = 0$. We set $a = \mu + \sigma_1$, then we have $\beta_1 I_1 S - aE_1 = 0$. We recall that $\beta_1 I_1 S = \Lambda - \mu S$, hence $(\Lambda - \mu S_1^*) - aE_1 = 0$. Then, $\frac{1}{a}(\Lambda - \mu S_1^*) = E_1$. Therefore, $E_1 = E_1^* = \frac{1}{a}(\Lambda - \mu S_1^*)$.

Considering the exposed compartment of strain 2, We set equation (13) to zero. Then we have $\beta_2 I_2 S - (\mu + \sigma_2)E_2 = 0$. If we consider $I_1 \neq 0$, and $I_2 = 0$ at the strain 1 endemic, $E_2 = E_2^* = 0$.

From equation (14), we set $\frac{dI_1}{dt} = 0$. Then we have $\sigma_1 E_1 - (\delta + \mu + \alpha_1 + \gamma_1)I_1 = 0$. Let $c = \delta + \mu + \alpha_1 + \gamma_1$, then $\sigma_1 E_1 - cI_1 = 0$. We recall that $E_1 = \frac{1}{a}(\Lambda - \mu S_1^*)$ and then $\frac{\sigma_1}{a}(\Lambda - \mu S_1^*) - cI_1 = 0$. Therefore $I_1 = \frac{\sigma_1}{ac}(\Lambda - \mu S_1^*)$.

From equation (15), we set $\frac{dI_2}{dt} = 0$. Then we have $\sigma_2 E_2 - (\delta + \mu + \alpha_2 + \gamma_2)I_2 = 0$. Then we obtain $\frac{\sigma_2 E_2}{\delta + \mu + \alpha_1 + \gamma_2} = I_2$. We recall that $E_2 = 0$, therefore we have $I_2 = 0$.

From equation (16), we set $\frac{dQ}{dt} = 0$. Then we have $\alpha_1 I_1 + \alpha_2 I_2 - (\mu + \theta)Q = 0$. If we consider $I_1 \neq 0$, and $I_2 = 0$, then $\alpha_1 I_1 - (\mu + \theta)Q = 0$. We recall that $I_1 = \frac{\sigma_1}{ac}(\Lambda - \mu S_1^*)$ then $\frac{\alpha_1 \sigma_1}{ac}(\Lambda - \mu S_1^*) - (\mu + \theta)Q = 0$. Therefore, $Q = Q^* = \frac{\alpha_1 \sigma_1}{ac(\mu + \theta)}(\Lambda - \mu S_1^*)$. At the endemic equilibrium for strain 1,

$$\varepsilon_1 = \left(S_1^*, \frac{1}{a}(\Lambda - \mu S_1^*), 0, \frac{\sigma_1}{ac}(\Lambda - \mu S_1^*), 0, \frac{\alpha_1 \sigma_1}{ac(\mu + \theta)}(\Lambda - \mu S_1^*)\right).$$

With regards to the strain 2 endemic equilibrium ($\varepsilon_2$), $I_1 = 0$ and $I_2 \neq 0$.
From equation (11), we set $\frac{dS}{dt} = 0$. Then we have $\Lambda - \beta_1 I_1 S - \beta_2 I_2 S - \mu S + \upsilon R = 0$. If we consider $I_1 = 0, I_2 \neq 0$, and $R = 0$, we have $\Lambda - \beta_2 I_2 S - \mu S = 0$. Then, $\Lambda - \mu S = \beta_2 I_2 S$. We further consider $\Lambda = \beta_1 I_1 S + \mu S$, then $\Lambda = (\beta_2 I_2 + \mu)S$. Therefore, $S = S_1^* = \frac{\Lambda}{\beta_2 I_2 + \mu}$

With regards to the exposed compartment of strain 1 (12), we set $\frac{dE_1}{dt} = 0$. Then we have $\beta_1 I_1 S - (\mu + \sigma_1)E_1 = 0$. If we consider $I_1 = 0$, and $I_2 \neq 0$ at the strain 2 endemic, $E_1 = E_1^* = 0$

From equation (13), we set $\frac{dE_2}{dt} = 0$. Then $\beta_2 I_2 S - (\mu + \sigma_2)E_2 = 0$. We set $b = \mu + \sigma_2$, then we have $\beta_2 I_2 S - bE_2 = 0$. We recall that $\beta_2 I_2 S = \Lambda - \mu S$, hence $(\Lambda - \mu S_2^*) - bE_1 = 0$. Then, $\frac{1}{b}(\Lambda - \mu S_2^*) = E_2$. Therefore, $E_2 = E_2^* = \frac{1}{b}(\Lambda - \mu S_2^*)$.

From equation (14), we set $\frac{dI_1}{dt} = 0$. Then we have $\sigma_1 E_1 - (\delta + \mu + \alpha_1 + \gamma_1)I_1 = 0$. And then $\frac{\sigma_1 E_1}{\delta + \mu + \alpha_1 + \gamma_1} = I_1$. We recall that $E_1 = 0$, therefore $I_1 = 0$.

From equation (15), we set $\frac{dI_2}{dt} = 0$. Then we have $\sigma_2 E_2 - (\delta + \mu + \alpha_2 + \gamma_2)I_2 = 0$. Let $d = \delta + \mu + \alpha_2 + \gamma_2$, then $\sigma_2 E_2 - dI_2 = 0$. We recall that $E_2 = \frac{1}{b}(\Lambda - \mu S_2^*)$ and then $\frac{\sigma_2}{b}(\Lambda - \mu S_2^*) - dI_2 = 0$. Therefore $I_2 = \frac{\sigma_2}{bd}(\Lambda - \mu S_2^*)$.

From equation (16), we set $\frac{dQ}{dt} = 0$. Then we have $\alpha_1 I_1 + \alpha_2 I_2 - (\mu+\theta)Q = 0$. If we consider $I_1 = 0$, and $I_2 \neq 0$, then $\alpha_2 I_2 - (\mu+\theta)Q = 0$. We recall that $I_2 = \frac{\sigma_2}{bd}(\Lambda - \mu S_2^*)$ then $\frac{\alpha_2\sigma_2}{ac}(\Lambda - \mu S_2^*) - (\mu+\theta)Q = 0$. Therefore, $Q = Q^* = \frac{\alpha_2\sigma_2}{bd(\mu+\theta)}(\Lambda - \mu S_2^*)$. At the endemic equilibrium for strain 2,

$$\varepsilon_2 = \left(S_2^*, 0, \frac{1}{b}(\Lambda - \mu S_2^*), 0, \frac{\sigma_2}{bd}(\Lambda - \mu S_2^*), \frac{\alpha_2\sigma_2}{bd(\mu+\theta)}(\Lambda - \mu S_2^*)\right).$$
