# Supplementary material for: A mathematical model of COVID-19 with multiple variants of the virus under optimal control in Ghana
Source: PLoS One. 2024 Jul 2;19(7):e0303791. doi: 10.1371/journal.pone.0303791 (PMC11218976; doi:10.1371/journal.pone.0303791)
Supplement: S2 Appendix — This appendix contains all combinations of the two control strategies. Therefore, it includes combinations of 1 and 2, 2 and 3, and 1 and 3 and their results analysis. (PDF) [file pone.0303791.s002.pdf]

1 Combination of two Control Strategies

1.1 Social Distancing and Vaccination

Here, we looked at the effect of two control strategies and their effects on COVID-19 pandemic. In figure 1, we considered the effect of control strategy 1 (social distancing) and control strategy 2 (vaccination) at a mild, average, and strict level while control strategy 3 was set at zero. Subfigure 1(a) and 1(b) showed the effect of the control strategies at a mild and strict level respectively, it was noticed that there was more than 50% reduction in the spread of infection when the control strategies were at a strict level. The social distancing, and vaccination at a strict level ($u_1, u_2 = 0.8, 0.8$) implemented at an early stage of the pandemic shows no outbreak, however this is impossible in the real world as a vaccine for a new disease has not been made.

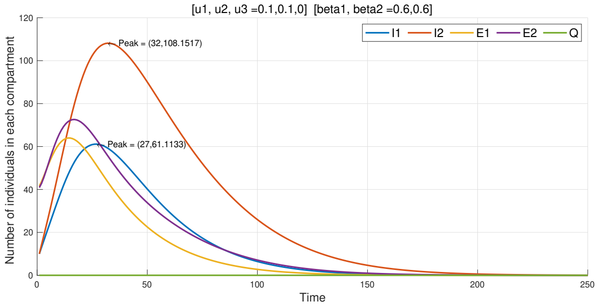

(a) Mild level for both u_1 and u_2 ($u_1, u_2 = 0.1, 0.1$)

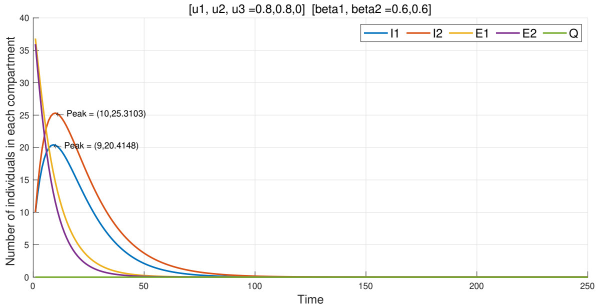

(b) Strict level for both u_1 and u_2 ($u_1, u_2 = 0.8, 0.8$)

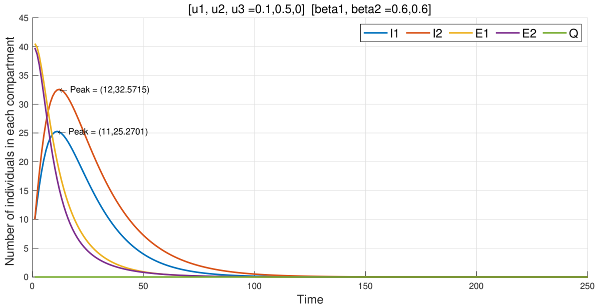

(c) u_1 is mild level, u_2 is average level ($u_1, u_2 = 0.1, 0.5$)

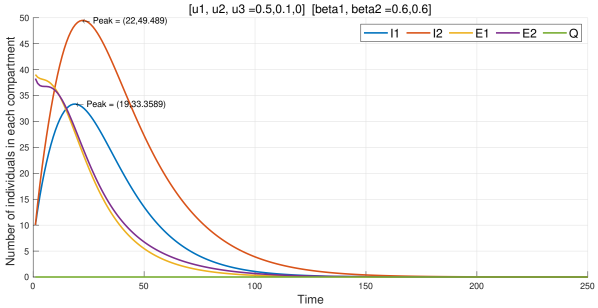

(d) u_1 is average level, u_2 is mild level ($u_1, u_2 = 0.5, 0.1$)

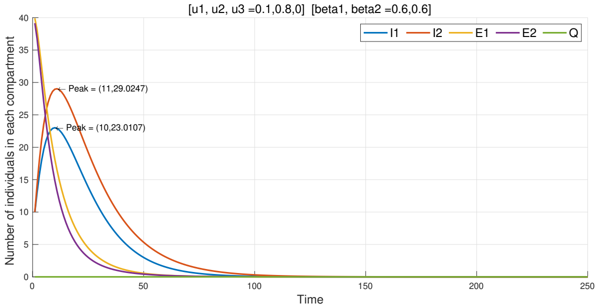

(e) u_1 is mild level, u_2 is strict level ($u_1, u_2 = 0.1, 0.8$)

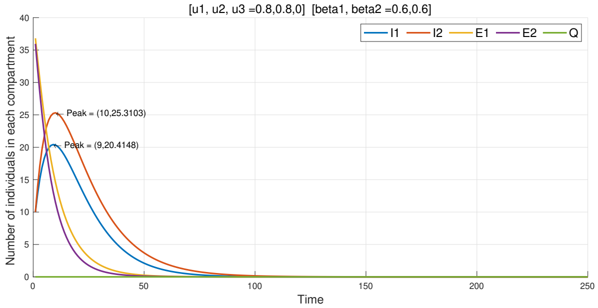

(f) u_1 is strict level, u_2 is mild level ($u_1, u_2 = 0.8, 0.1$)

Figure 1: Control Strategy 1 & 2.

Also, the effect of the combination of u_1 and u_2 was viewed on the individual parameters. When u_1 and u_2 were implemented, there were 61 individuals infected with the original virus having its peak at 27 days for a mild level while at a strict level there were only 9 individuals infected with its peak at 20 days at a mild and strict level respectively. With regards to I_2, there were 108 individuals infected with the mutant virus having a peak of 32 days and 25 individuals with a peak of 10 days at a mild and strict level respectively. With regards to the exposed compartment, E_1 had 62 individuals exposed to the original virus while there were 70 individuals exposed to the virus when u_1 and u_2 are at a mild

level 1(a) and at a strict level E_1 and E_2 had no outbreak which could be due to the individuals that are immune to the virus after vaccination coupled with the social distancing.

The control strategies u_1 and u_2 were also considered at different levels to determine their respective effect on the reduction of the COVID-19 disease. In subfigure 1(c), u_1 was at a mild level while u_2 was at an average level, this was in contrast with subfigure 1(d). Regarding I_1 when u_1 at a mild level and u_2 at an average level, there were 25 individuals infected with the virus having a peak of 11 days compared to when u_1 is at an average level and u_2 at a mild level with 34 individuals infected and a peak of 19 days. Similarly, I_2 when u_1 at a mild level and u_2 at an average level, there were 25 individuals infected with the virus having a peak of 11 days compared to when u_1 is at an average level and u_2 at a mild level with 48 individuals infected and a peak of 22 days. It was further observed that there was no outbreak with regards to E_1, E_2 at subfigure 1(c) and 1(d). The result suggested that u_1 at a mild level and u_2 at an average level were more effective in reducing the number of infected individuals compared to u_1 at an average level and u_2 at a mild level.

Additionally, we considered u_1 at a mild level and u_2 1(e) at a strict level in contrast with u_1 at a strict level and u_2 at a mild level 1(f). Regarding I_1 when u_1 at a mild level and u_2 at a strict level, there were 23 individuals infected with the virus having a peak of 10 days compared to when u_1 is at a strict level and u_2 at a mild level with 23 individuals infected and a peak of 12 days. Similarly, I_2 when u_1 at a mild level and u_2 at a strict level, there were 29 individuals infected with the virus having a peak of 11 days compared to when u_1 is at a strict level and u_2 at a mild level with 30 individuals infected and a peak of 14 days. It was further observed that there was no outbreak with regards to E_1, E_2 at subfigure 1(e) and 1(f). The result suggested that u_1 at a mild level and u_2 at a strict level were more effective in reducing the number of infected individuals compared to u_1 at a strict level and u_2 at a mild level.

It was observed that u_1 at a mild level and u_2 1(e) at a strict level and u_1 at a strict level and u_2 1(f) at a mild level results were similar to the comparison between1(c) and 1(d); however, there was little difference in its effectiveness. Finally, it could be seen that u_2 (vaccination) was more effective in reducing the spread of the disease as compared to u_1 (social distancing).

The analysis of the control 1 (social distancing) and control 2 (vaccination) strategies is summarized in table 1 as follows:

Table 1: Control 1 & 2 Strategy

Control 1 & 2	I_1	I_2	E_1	E_2	Q
$u_1, u_2 =$	61 (I)	108 (I)	62 (I)	70 (I)	No effect
$0.1, 0.1$	27 (P)	32 (P)	15(P)	18(P)	
$u_1, u_2 =$	9 (I)	25 (I)	No outbreak	No outbreak	
$0.8, 0.8$	20 (P)	10 (P)			
$u_1, u_2 =$	25 (I)	25 (I)			
$0.1, 0.5$	11 (P)	11 (P)			
$u_1, u_2 =$	34 (I)	48 (I)			
$0.5, 0.1$	19 (P)	22 (P)			
$u_1, u_2 =$	23 (I)	29 (I)			
$0.1, 0.8$	10 (P)	11 (P)			
$u_1, u_2 =$	23 (I)	30 (I)			
$0.8, 0.1$	12 (P)	14 (P)			
$I =$ No of Individuals $P =$ Peak (days)					

Table notes the number of individuals at the peak of each compartment of the mixed strategy of optimal control strategies 1 and 2 and the period until the peak occurred. 'No outbreak' signifies compartments without significant outbreaks.

1.2 Social Distancing and Testing-treatment

In figure 2, we considered control strategy 1 (social distancing) and control strategy 3 (testing-treatment of COVID-19 patients) at a mild, average, and strict level. Subfigure 2(a) and 2(b) showed the effect

of the control strategies at a mild and strict level respectively. It was observed that u_1 and u_3 at a strict level lead to a great reduction of the disease compared to being at a mild level.

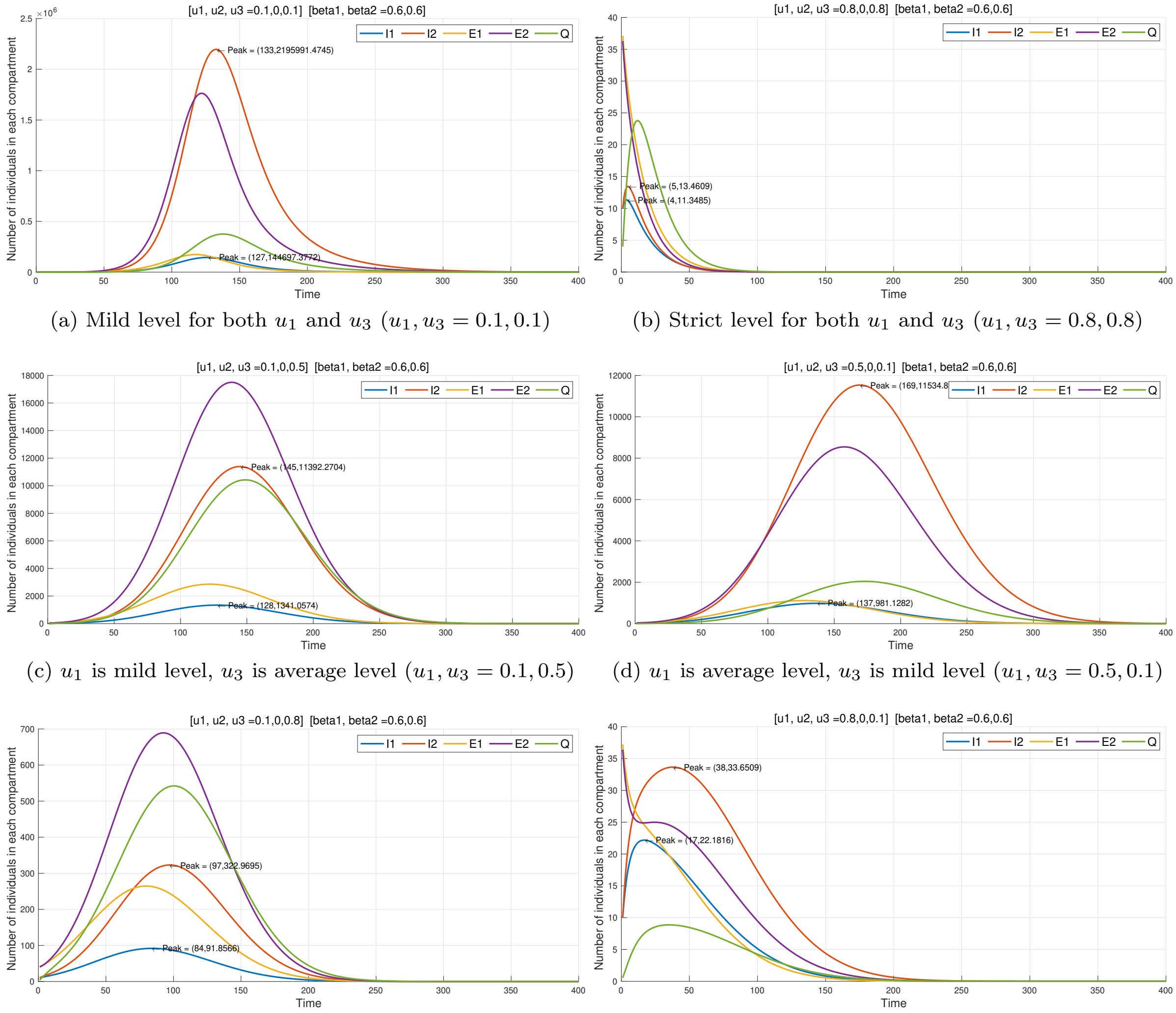

(a) Mild level for both u_1 and u_3 $(u_1, u_3 = 0.1, 0.1)$

(b) Strict level for both u_1 and u_3 $(u_1, u_3 = 0.8, 0.8)$

(c) u_1 is mild level, u_3 is average level $(u_1, u_3 = 0.1, 0.5)$

(d) u_1 is average level, u_3 is mild level $(u_1, u_3 = 0.5, 0.1)$

(e) u_1 is mild level, u_3 is strict level $(u_1, u_3 = 0.1, 0.8)$

(f) u_1 is strict level, u_3 is mild level $(u_1, u_3 = 0.8, 0.1)$

Figure 2: Control Strategy 1 & 3.

Furthermore, we viewed the effect of u_1 and u_3 was considered on the individual compartments. With respect to when u_1 and u_3 were implemented, there were 144,697 individuals infected with the original and delta virus (I_1) having its peak at 127 days for a mild level while at a strict level there were only 11 individuals infected with it peak at 4 days at a mild and strict level respectively. while I_2 had 2,195,991 individuals infected with a peak of 133 days at a mild level and 13 individuals infected with a peak of 5 days at a strict level.

Additionally, we considered exposed individuals (E_1, E_2) at both levels; a lot of individuals were exposed to the COVID-19 virus at a mild level meanwhile there were no outbreaks at a strict level. With regards to the quarantine compartment, few individuals were quarantined at a mild level as little to no control was implemented while at the strict level, individuals who got exposed and infected with the disease went into quarantine to prevent the further spread of the disease.

The combination of u_1 and u_3 at a mild level is the worst combination of the control strategies to be implemented as a lot of people are exposed to the virus and get infected without caring about the social distancing, and testing-treatment of COVID-19 virus.

Likewise, we compared u_1 and u_3 2(c) at a mild level and average level respectively with u_1 and u_3 2(d) at an average level and mild level respectively. Regarding I_1 when u_1 at a mild level and u_3 at an average level, there were 1,341 individuals infected with the virus having a peak of 128 days

compared to when u_1 is at an average level and u_3 at a mild level with 981 individuals infected and a peak of 137 days. Similarly, I_2 when u_1 at a mild level and u_3 at an average level, there were 11,392 individuals infected with the virus having a peak of 145 days compared to when u_1 is at an average level and u_3 at a mild level with 11,534 individuals infected and a peak of 169 days. The exposed compartments (E_1, E_2) reduced slightly when u_1 was at an average level and u_3 at a mild level as compared to when u_1 was at a mild level and u_3 at an average level. With regards to quarantine, there were more individuals who were quarantined (10,000) when u_1 was at a mild level and u_3 at an average level as compared to when u_1 was at an average level and u_3 at a mild level (2,000). It was observed that the infection rates were at a minimum when $u_1 = 0.5$ and $u_3 = 0.1$ while the exposed rates were higher when $u_1 = 0.1$ and $u_3 = 0.5$.

Finally, in the mild and strict combination of the control strategies, subfigure 2(f) showed a significant reduction in the number of exposed and infected people when $u_1 = 0.8$ and $u_3 = 0.1$ as compared to 2(e) when $u_1 = 0.1$ and $u_3 = 0.8$. The analysis of control 1 (social distancing) and control 3 (testing-treatment of COVID-19 patients) strategies is summarized in table 2.

Table 2: Control 1 & 3 Strategy

<table>
<tr><th>Control 1 & 3</th><th>I_1</th><th>I_2</th><th>E_1</th><th>E_2</th><th>Q</th></tr>
<tr><td rowspan="2">$u_1, u_3 =$ $0.1, 0.1$</td><td>144,697 (I)</td><td>2,195,991 (I)</td><td>62 (I)</td><td>70 (I)</td><td>61 (I)</td></tr>
<tr><td>127 (P)</td><td>133 (P)</td><td>27 (P)</td><td>27(P)</td><td>27 (P)</td></tr>
<tr><td rowspan="2">$u_1, u_3 =$ $0.8, 0.8$</td><td>11 (I)</td><td>13 (I)</td><td rowspan="2">No outbreak</td><td rowspan="2">No outbreak</td><td>61(I)</td></tr>
<tr><td>4 (P)</td><td>5 (P)</td><td>27 (P)</td></tr>
<tr><td rowspan="2">$u_1, u_3 =$ $0.1, 0.5$</td><td>1,341 (I)</td><td>11,392 (I)</td><td>2,500 (I)</td><td>17,000 (I)</td><td>10,000 (I)</td></tr>
<tr><td>128 (P)</td><td>145 (P)</td><td>125 (P)</td><td>142 (P)</td><td>150 (P)</td></tr>
<tr><td rowspan="2">$u_1, u_3 =$ $0.5, 0.1$</td><td>981 (I)</td><td>11,534 (I)</td><td>1000 (I)</td><td>8200 (I)</td><td>2,000 (I)</td></tr>
<tr><td>137 (P)</td><td>169 (P)</td><td>No outbreak</td><td>155 (P)</td><td>175 (P)</td></tr>
<tr><td rowspan="2">$u_1, u_3 =$ $0.1, 0.8$</td><td>98 (I)</td><td>310 (I)</td><td>200 (I)</td><td>695 (I)</td><td>550 (I)</td></tr>
<tr><td>84 (P)</td><td>97 (P)</td><td>60 (P)</td><td>98 (P)</td><td>100 (P)</td></tr>
<tr><td rowspan="2">$u_1, u_3 =$ $0.8, 0.1$</td><td>22 (I)</td><td>33 (I)</td><td rowspan="2">No outbreak</td><td rowspan="2">No outbreak</td><td rowspan="2">No outbreak</td></tr>
<tr><td>17 (P)</td><td>38 (P)</td></tr>
<tr><td colspan="6">I = No of Individuals, P = Peak (days)</td></tr>
</table>

1.3 Vaccination and Testing-treatment

In figure 3, we considered control strategy 2 (vaccination) and control strategy 3 (testing-treatment of COVID-19 patients) at a mild, average, and strict level. Subfigure 3(a) and 3(b) showed the effect of the control strategies at a mild and strict level respectively. It was observed that u_2 and u_3 at a strict level lead to a reduction of the disease compared to being at a mild level.

However, quarantine at the mild level is lesser which could be due to less screening, testing-treatment of COVID-19 patients as well as fewer people getting vaccinated. Also, we compared u_2 and u_3 3(c) at a mild level and average level respectively with u_2 and u_3 3(d) at an average level and mild level respectively. At 3(c), showed more people were quarantined as there were more screening, testing-treatment of COVID-19 done as compared to 3(d) which had more people vaccinated. Subfigure 3(d) showed that diseases were at a minimal which was in line with literature that COVID-19 vaccination was associated with a 90% reduction in risk for severe COVID-19 outcomes. In subfigure 3(e), there was an increase in the disease as more people got tested and became aware of been infected or not, thereby leading to treatment of the diseases and getting quarantined while in subfigure 3(f), there was less infection and spread of the disease due to a strict vaccination policy.

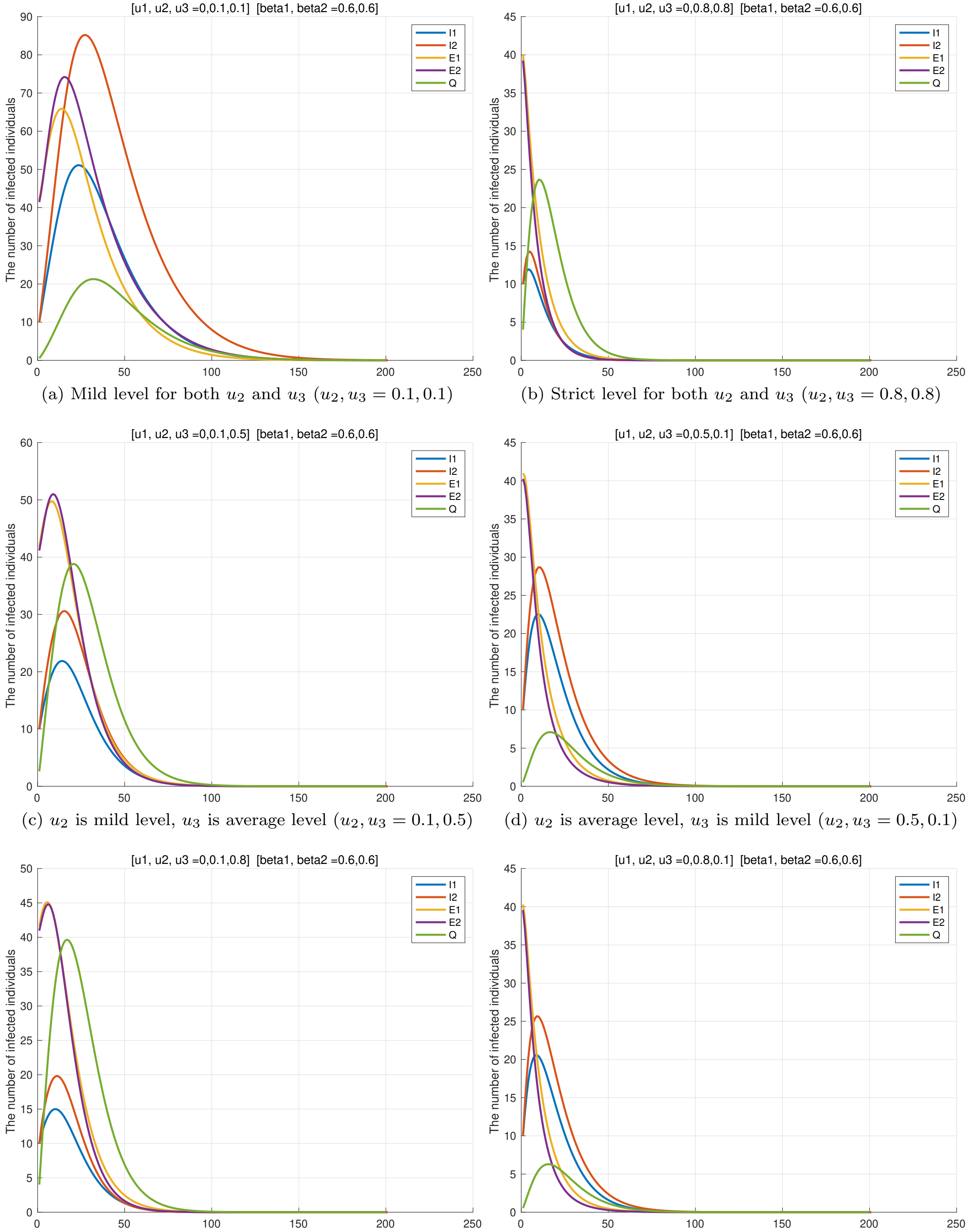

(a) Mild level for both u_2 and u_3 $(u_2, u_3 = 0.1, 0.1)$

(b) Strict level for both u_2 and u_3 $(u_2, u_3 = 0.8, 0.8)$

(c) u_2 is mild level, u_3 is average level $(u_2, u_3 = 0.1, 0.5)$

(d) u_2 is average level, u_3 is mild level $(u_2, u_3 = 0.5, 0.1)$

(e) u_2 is mild level, u_3 is strict level $(u_2, u_3 = 0.1, 0.8)$

(f) u_2 is strict level, u_3 is mild level $(u_2, u_3 = 0.8, 0.1)$

Figure 3: Control Strategy 2 & 3.
