# Supplementary material for: A mathematical model of COVID-19 with multiple variants of the virus under optimal control in Ghana
Source: PLoS One. 2024 Jul 2;19(7):e0303791. doi: 10.1371/journal.pone.0303791 (PMC11218976; doi:10.1371/journal.pone.0303791)
Supplement: S1 File — This material includes the data from Ghana we used and the code written in MATLAB to obtain data fitting results and optimal control results. (ZIP) [file pone.0303791.s003.zip › code_matlab/data/Noguchi Covid 19 research.pdf]

Preprints (www.preprints.org) | NOT PEER-REVIEWED | Posted: 26 September 2022 doi:10.20944/preprints202209.0390.v1

Article

Determination of SARS-CoV-2 Variants and Seroprevalence in Accra, Ghana during the Delta-Omicron Waves

Irene Owusu Donkor[1*], Elvis S. Lomotey[2], Jewelna Akorli[2], Millicent Opoku[2], Emmanuel Frimpong Gyekye[1], Kojo Mensah Sedzro[1], Nana Efua Andoh[2], Yvonne Ashong[2], Benjamin Abuaku[1], Kwadwo A. Koram[1] and Vincent Munster[3]

[1] Department of Epidemiology, Noguchi Memorial Institute for Medical Research, University of Ghana, Legon
[2] Department of Parasitology, Noguchi Memorial Institute for Medical Research, University of Ghana, Legon
[3] Virus Ecology Section, Laboratory of Virology, Rocky Mountain Laboratories, National Institute of Allergy and Infectious Diseases, National Institutes of Health, Hamilton, USA
*Corresponding author: iowusu@noguchi.ug.edu.gh

Abstract: A significant proportion of SARS-CoV-2 infections in Africa are identified as asymptomatic, facilitating the silent spread of the virus especially in populated urban cities. With the surge of the highly transmissible Omicron variant, the inclusion of asymptomatics in epidemiological surveys is key in estimating true infections and seroprevalence in the population. The aim of the study was to determine seroprevalence, active infection and circulating variants in Accra, the capital city of Ghana during the Omicron wave. The study was a cross-sectional survey conducted in 22 municipalities in December 2021. Naso-oropharyngeal swabs and serum samples were collected from 1027 individuals aged 5 years and above, for detection of infection by RT-qPCR and estimation of total antibodies using the WANTAI ELISA kit. Our results show 10% SARS-CoV-2 prevalence, with the Omicron and Delta variants accounting for 44.1% and 8.8% of infections, respectively. Omicron was most prevalent (48.9.%) among the 20–39-year-olds. Asymptomatic individuals accounted for 75.2% of infections. Seropositivity within the population was 86.8%, with the 60+ year group having significantly higher likelihood of exposure (OR 10.22: 95% CI: 3.51-29.73; $p<0.001$). This high seroprevalence appears to have been as a result of increased vaccination among this group (OR 2.7: 95% CI 1.78-4.09, $p < 0.001$). The high seropositivity of SARS-CoV-2 in the capital could be a good indication of herd immunity among the population and while the low infection rate supports the role of vaccination in reducing viral transmission.

Keywords: SARS-CoV-2; COVID-19; asymptomatic; seroprevalence; Delta variant; Omicron variant; vaccination

1. Introduction

The Severe Acute Respiratory Syndrome Coronavirus-2 (SARS-CoV-2) has been associated with over a million deaths since its emergence in 2019 [1, 2]. Although restrictions have been significantly eased globally, active infections continue to occur [3, 4]. The African continent recorded the least number of cases and mortality over the 2-year period when the pandemic was at its peak [5, 6]. According to WHO reports, two-thirds of the African population is exposed to SARS-CoV-2 with more than 60% of cases being asymptomatic [7–10].

Based on the trajectory of recorded deaths and the relative contagiousness of distinct SARS-CoV-2 variants, the progression of the pandemic in Africa has been shown to differ from other parts of the world [5]. The successive waves of the virus have been classified by ‘variants of concern’ which have by far superseded the wildtype strain. The Delta and Omicron variants have been the most dominant variants in Africa [11]. Studies have indicated that, the high transmissibility of the Omicron variant has been coupled with

© 2022 by the author(s). Distributed under a Creative Commons CC BY license.

minimal severity of illness globally[11, 12] as compared to the Delta variant which is associated with a higher disease burden [13]

The first confirmed cases of COVID-19 in Ghana were recorded in March 2020, soon after the WHO declaration of a global pandemic. The disease spread quickly through the population with majority of cases identified in the two most populated cities in Ghana, Accra and Kumasi [14]. These areas recorded high numbers of asymptomatic individuals who had no travel history, indicating active local transmission which led to several restrictions to reduce spread [9, 15]. A seroprevalence study in the last and first quarter of 2020 and 2021, respectively, showed increasing seropositivity in two selected regions of the country [15]. Thereafter, significant COVID-19-related events occurred including the emergence of more transmissible variants of the virus i.e Delta and Omicron, and vaccination against SARS-CoV-2 which are expected to further increase seropositivity.

Evaluation of infection dynamics and the population disease burden remains a significant research focus by the World Health Organization to characterize key epidemiological features [16]. This study sought to estimate the extent of infection, seroprevalence and circulating variants of concern in Accra, the capital of Ghana and one of the highest disease burden areas for COVID-19, during the period when Ghana experienced its third wave of infections. We also assessed the relationships between vaccination and infection status of the study population and related this to the observed seroprevalence.

2. Materials and Methods

Study Design

This study was a cross-sectional survey targeting 22 selected municipalities in Accra, in December 2021. The survey selected households in Enumeration Areas (EAs) from the National Sampling Frame obtained from the Ghana Statistical Service. Using the Kish selection grid and left-hand rule [17], 20 households were selected in each community. According to our selection method, one participant from each household was randomly chosen to represent a group in the following age stratifications: 5-9, 10-19, 20-39, 40-59, or 60+ years. Consent was first sought from the head of each household, where required. Adult consent (in addition to parental consent in the case of minors) was obtained from participants aged 18 years, parental consent from participants aged less than 18 years, and child assent from those aged 12-17 years. Sociodemographic information, vaccination and health status were obtained from all consenting participants. Adherence to WHO COVID-19 guidelines was also assessed using a structured questionnaire.

Sample Collection

Naso and oropharyngeal swabs were collected from each participant according to Standard Operating Procedures (SOPs) [18] and transferred to viral transport medium (VTM). The samples were transported in cold chain to the laboratory. A total of 5 ml whole blood was also collected from each participant following the WHO guidelines on venipuncture [19] and divided into a serum separator and EDTA tubes. The serum separator tubes and swabs were stored on ice. All samples were transported to the laboratory at the Noguchi Memorial Institute for Medical Research (NMIMR), University of Ghana for processing and laboratory analysis [20]. All samples were processed the same day.

Molecular detection of SARS-COV-2

Viral RNA was extracted from swabs in 96-well plates with the QIAamp Viral RNA Mini Kit (Qiagen Str, Hilden, Germany) using a vacuum pump extractor. The TIB MOLBIOL (Berlin Germany) LightMix® SARS-CoV-2 E+N UBC Kit and the LightMix® Modular SARS-CoV-2 (COVID-19) RdRP Gene Kit complemented with Luna® Universal qPCR Master Mix, were used for the amplification and quantitative detection of SARS-CoV-2, respectively. The detection of the virus in a sample was a two-step process involving a screening assay targeting the SARS-CoV-2 E and N genes and confirmatory assay targeting the RNA-dependent RNA polymerase gene (RdRp), a gene specific to SARS-

Preprints (www.preprints.org) | NOT PEER-REVIEWED | Posted: 26 September 2022 doi:10.20944/preprints202209.0390.v1

CoV-2. Quality controls provided in the TIB MOLBIOL kit were included in all assay plates. Controls included a positive Equine Arteritis Virus (EAV) extraction control (ref. 40-0776-96) to detect possible inhibition of PCR, positive controls for E gene (ref. 40-0776-96), RdRp gene (ref. 53-0777-96) and a no-template control (NTC) to detect contamination. The validity of the test was only accepted if the cycle threshold (Ct) value of the E+N and RdRp positive controls was < 36, that for the EAV was < 33 and if the NTC did not generate an amplification curve. A sample was considered conclusively positive in valid tests if the Ct values of E+N and RdRp are < 36. If the RdRp gene was not observed or the Ct > 40, the sample was considered probable for SARS virus. A sample was considered negative if no amplification curve was observed for the E+N and RdRp genes.

Molecular detection of Delta and Omicron variants

SARS-CoV-2 E-Spike Delta/Omicron TaqMan Typing kit (TIBMOLBIOL: ref 40-0811-96) was used according to the manufacturer's protocol to detect the SARS Spike ins214EPE and SARS Spike del157/158 specific to Omicron and Delta variants, respectively. A human mRNA sample (Ubiquitin C) was provided as internal control for the assay. Amplifications (< 35) at the FAM and HEX detection channels were accepted as Omicron and Delta positives, respectively, while those at the ROX channel were considered as 'other variants'.

Detection of antibodies against SARS-CoV-2

The WHO WANTAI SARS-CoV-2-Ab kit (ref. WS-1096), a sandwich Enzyme Linked Immunosorbent Assay (ELISA), was used for the qualitative detection of total antibodies to SARS-CoV-2 in each processed serum specimen. This is a two-step procedure using pre-coated recombinant SARS-CoV-2 antigen to capture specific SARS-CoV-2 antibodies in the serum if present. A second recombinant SARS-CoV-2 antigen conjugated to the enzyme Horseradish Peroxidase (HRP-Conjugate) is added to the immunocomplex. The absorbance of detectable antibody captured in the complete immunocomplex was measured with a BioTek® Microplate Reader (Gen 5 3.10). The results were calculated by relating each specimen absorbance value to the cut-off value of the test plate. The cut-off reading is based on single filter plate reader. First, the absorbance value in the blank well was subtracted from the absorbance value of test samples and controls. The cut-off (CO) value was then calculated by adding 0.16 (constant) as stated in the manufacturer's protocol to the mean absorbance of the negative controls (NC). The ratio of the absorbance of the test samples to the cut-off value determined the presence or absence of SARS-CoV-2 total antibodies. Specimen with antibody detection value >1 were considered reactive, which implied the presence of SARS-CoV-2 antibodies. Specimen with antibody detection value < 1 were considered negative i.e. no serological indication for current or past coronavirus disease.

Data Analysis

Data collected was cleaned to check for duplications, errors and completeness using Microsoft Excel. The cleaned data was exported, coded, and analyzed using STATA 16 (StataCorp, College Station, TX, USA). GraphPad prism v9.3.1(Graph Pad Software, LLC) was further used for the representation of figures. Frequency and percentages were used for the descriptive analysis. Skewness test was performed to determine the normality of continuous variables. Pearson Chi Square tests and Mann Whitney U tests were performed to determine the association between seroprevalence and the factor variables. Logistic regression analysis was performed between SARS-CoV-2 infection prevalence, seroprevalence and the predictive variables to determine the exact groups within the categorical variables showing associations. A composite adherence score was computed based on nine self-reported observations of the WHO standard guidelines. To distinguish between low, moderate, and high levels of adherence, we developed a 4-level response category with variables of 0 indicating no adherence, < 2 indicating low adherence, 3-6

Preprints (www.preprints.org) | NOT PEER-REVIEWED | Posted: 26 September 2022 doi:10.20944/preprints202209.0390.v1

suggesting moderate adherence and > 7 indicating high adherence. Vaccination was compared from 18 years and above. This is because at the time of the study, eligible individuals for vaccination against SARS-CoV-2 were the aforementioned aged groups. Statistical inferences were made at p values at 0.05 and below with a 95% confidence interval.

3. Results

Prevalence of SARS-CoV-2 infection

A total of 1027 participants were sampled from 22 districts in Accra and screened for SARS-CoV-2 (Table S1). The overall infection prevalence among the study population was 10% (105/1027). The risk of infection was significantly high in the Ashiedu Keteke Municipality (OR 4.09; 95% CI 1.63-10.28, p=0.003) where the highest infection prevalence (82%) was also recorded (Fig 1) as compared with Ablekuma Central (Table 1). Mean prevalence of SARS-CoV-2 infection did not differ between gender (χ^2=0.44; p=0.51) or among the age groups (χ^2=7.81; p=0.25).

Table 1. SARS-CoV-2 infection among participant in the selected study areas. District/municipality where significant odds of finding SARS-CoV-2 positivity is indicated in bold.

District/Municipality	SARS-CoV-2 Negative	SARS-CoV-2 Positive	OR (95%CI)	*p*-value
Ablekuma Central	50	10	Ref	
Ablekuma South	39	2	0.26 (0.05-1.23)	0.090
Ada West	19	1	0.26 (0.03-2.20)	0.218
Ashaiman	69	10	0.72 (0.28-1.87)	0.506
**Ashiedu Keteke**	**22**	**18**	**4.09 (1.63-10.28)**	**0.003**
Ayawaso East	34	6	0.88 (0.29-2.66)	0.824
Ga Central	19	1	0.26 (0.03-2.20)	0.218
Ga South	129	11	0.43 (0.17-1.07)	0.068
La Dadekotopon	54	6	0.56 (0.19-1.64)	0.287
La Nkwantanan	14	6	2.14 (0.66-6.92)	0.203
Ledzokuku Krowor	108	12	0.56 (0.23-1.37)	0.202
Okai Koi South	28	12	2.14 (0.82-5.59)	0.119
Osu Klotey	14	7	2.5 (0.81-7.76)	0.113
Tema East	37	3	0.41 (0.10-1.58)	0.193

The variant detection kit used allowed for identification of Omicron and Delta variants for 51% (54/105) of the positive samples, implying that the remainder were infected with other variants. The Omicron variant accounted for 42.9% while the Delta variant was found in 8.6% of persons with SARS-CoV-2 (Fig. 2A). There was no significant difference in the distribution of the variants among males and females, or age groups (p> 0.05) (Fig. 2B).

Majority (75%) of the participants positive with SARS-CoV-2 showed no symptoms. The remaining 25% had between 1-3 symptoms (Fig. 3) with the most common being fever, chills, and headache. There was no association between the symptoms reported and the variants detected in positive participants (χ^2=7.32; p=0.12).

Preprints (www.preprints.org) | NOT PEER-REVIEWED | Posted: 26 September 2022 doi:10.20944/preprints202209.0390.v1

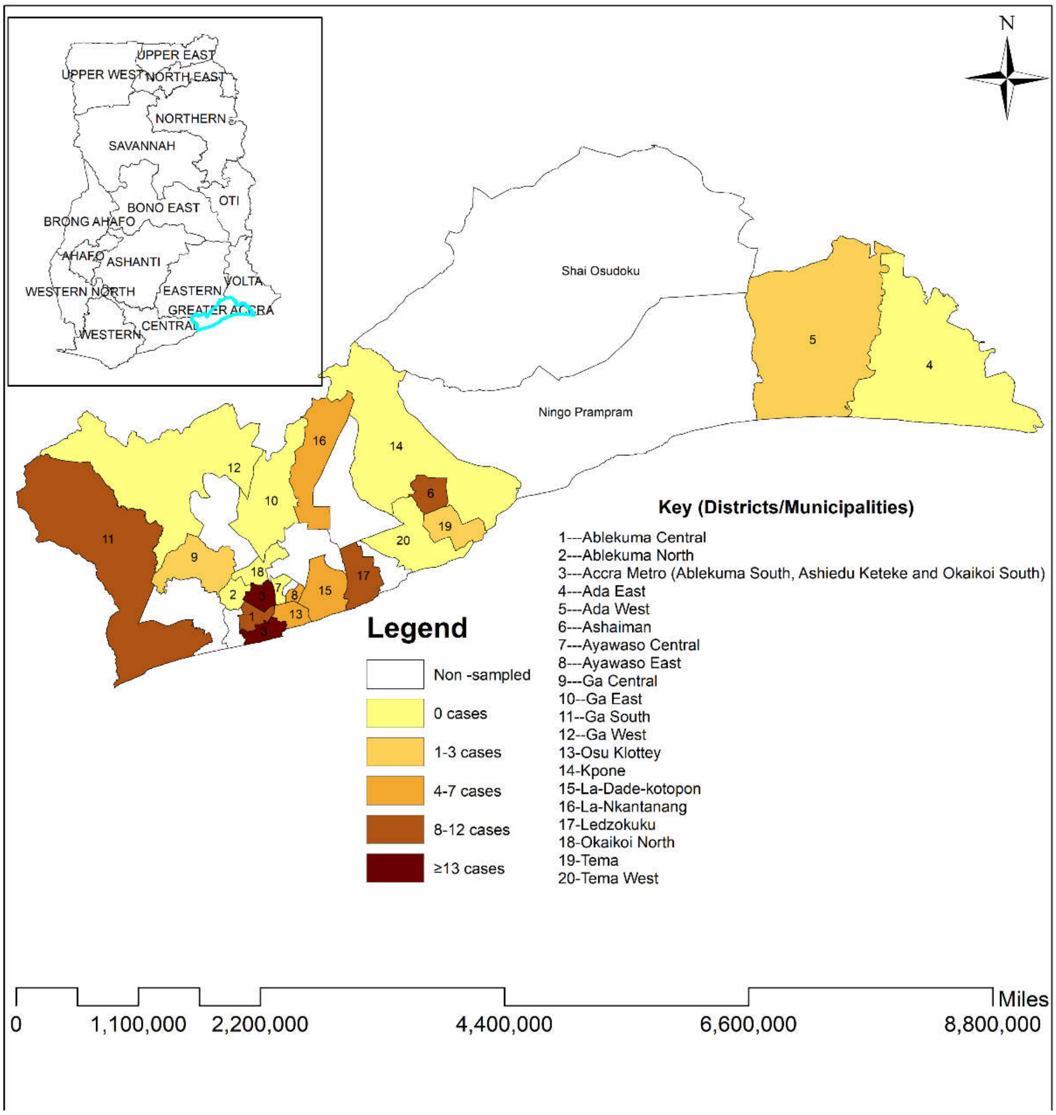

Figure 1. Distribution of SARS-CoV-2 infection in the study area. Prevalence map (A) shows the total number of positive individuals in the district/municipality. A total of 22 municipalities were sampled.

Preprints (www.preprints.org) | NOT PEER-REVIEWED | Posted: 26 September 2022 doi:10.20944/preprints202209.0390.v1

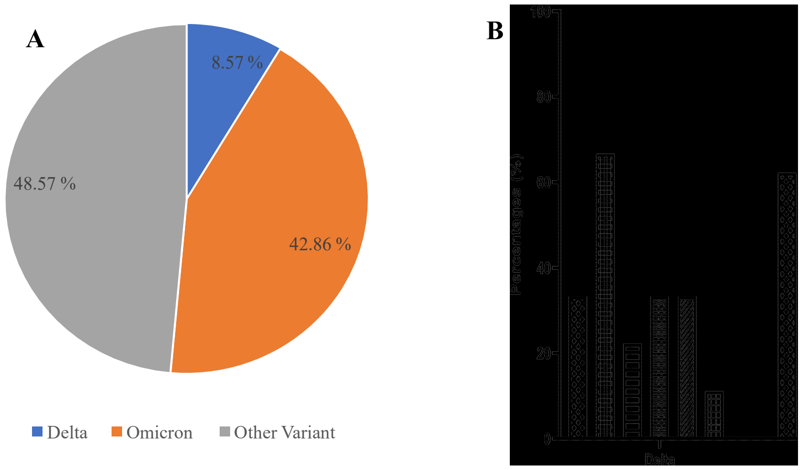

Figure 2. Distribution of SARS-CoV-2 variants among positive individuals in the study population. The total number of positives is 105. The pie chart (A) shows the frequency of Delta, Omicron and 'other variants' and bar plots (B) shows the prevalence of the circulating variants by sex and age groups. .

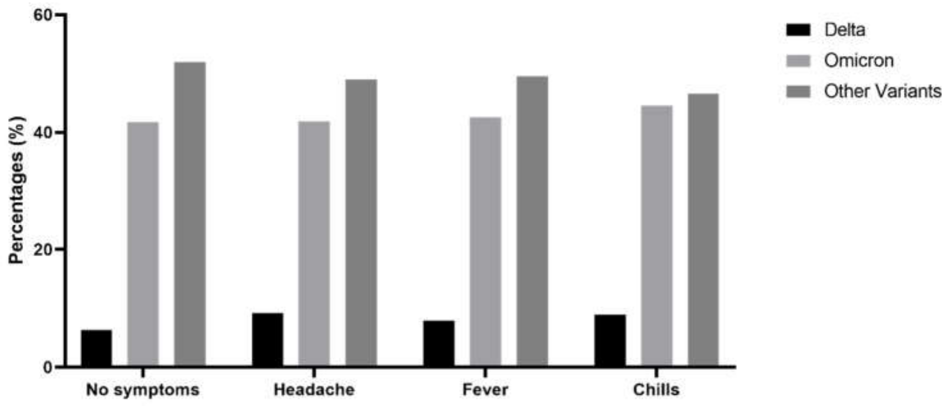

Figure 3. Reported symptoms among SARS-CoV-2 infected individuals. The symptoms were investigated for their association with the detected variants. None was detected (P= 0.12).

SARS-CoV-2 seroprevalence

We detected 86.8% overall prevalence of SARS-CoV-2 total antibodies in the study population and similar frequency between antigen-positive (85.7%) and negative (86.9%) individuals. The 60+ year group showed the highest likelihood of being seropositive (OR 10.22: 95% CI: 3.51-29.73; p< 0.001) although it is not clear whether this was due to previous exposure or vaccination. However, this same group showed higher possibility of having received vaccination (OR 4.31: 95% CI: 2.35-7.90, p< 0.001) (Table 2). There were no differences observed in the calculated average relative quantities of total antibodies between male and females or the different age groups (Mann-Whitney: p>0.05). Unvaccinated individuals showing antibodies against SARS-CoV-2 had lower average levels compared to those vaccinated (Mann-Whitney: p=0.04) (Fig. 4, Table 3).

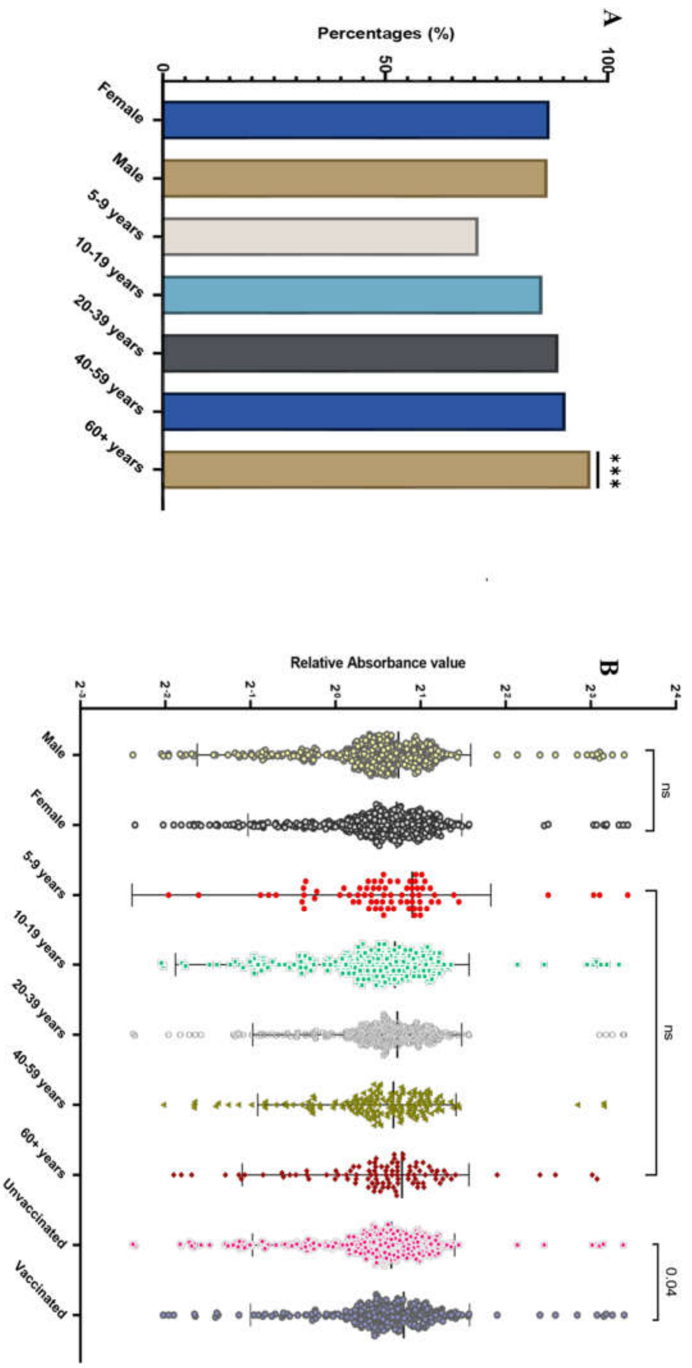

Figure 4. Seropositivity for SARS-CoV-2 total antibodies among the study groups. Bar plots (A) shows the percentage prevalence of antibodies for each group. Relative absorbance (B) values are shown for each group based on the total number of samples collected for that group and the number that show presence of antibodies.

Adherence to COVID-19 protocols

During the conduct of the study, we asked participants about their adherence to COVID-19 protocols in a guided questionnaire (Table 4). Frequent handwashing was the most practiced COVID-19 prevention activity (62%) among participants, followed by wearing of nose masks (45%). The adherence to the protocols were assessed in relation to infection status and risk of infection. All practices, apart from handwashing, self-isolating at home, avoiding public transport and working from home, showed significant associations with status of infection (Table 2). In most of these instances, there was significantly more positive individuals who confirmed to practicing infection prevention protocols. High adherence i.e. > 7 of the listed WHO standard guidelines was associated with

Preprints (www.preprints.org) | NOT PEER-REVIEWED | Posted: 26 September 2022 doi:10.20944/preprints202209.0390.v1

marginal significance in reducing the likelihood of COVID-19 infection (OR 0.56: 95% CI 0.32-0.99, p=0.045) while low adherence (< 2 of the of the WHO guidelines) to the protocols increased the risk of getting infected, although not significant (OR 1.27: 95% CI 0.74-2.20, p=0.39).

4. Discussion

This study was conducted to complement epidemiological data on COVID-19 in Ghana, especially during the Omicron infection wave and after several months of initiating vaccination against the disease. This was a community-based surveillance conducted in Accra, which has been established as one of the cities with high burden of SARS-CoV-2 [9]. We found an overall SARS-CoV-2 infection prevalence of 10% among the study population. In comparison, Europe and the Americas continued to report highest incidence of SARS-CoV-2 infection although there was reported decrease in the number of deaths at the time of the study in the African region at a steady peak of infection prevalence.[21]. The positivity rates were comparable between males and females, although other studies have reported higher infections in males [22, 23].

The SARS-CoV-2 variants of concern have impacted on the virus transmissibility and pathogenicity as well as vaccine effectiveness [24, 25]. Evidence in countries with documented transmission and high levels of population immunity suggests that Omicron variant has a growth advantage over the Delta. It remains uncertain to what extent the rapid growth rate can be attributed to immune evasion, intrinsic increased transmissibility, or a combination of both.[21]However, [26] recent evidence confirms the omicron variant emerged because of its ability to evade human preexisting immunity. A high prevalence of Omicron was expected since the study was conducted in a period when this variant wave was occurring. However, it was surprising that almost half (47%) of the infected participants had 'other variants'. This suggests that new variants do not rapidly replace existing ones and the latter are likely to be in circulation long enough to create new variants through recombination events. A limitation here was our incapacity to explore the other circulating variants detected in the study population and how those are associated with the parameters investigated in this study. Omicron was nonetheless high among 20-39 and 10-19 year-olds, supporting reports of high transmission among the work force [23]. This information also suggests the need to extend and encourage vaccination in Ghana to people in these age groups.

The high prevalence of SARS-CoV-2 total antibodies detected in our study are similar to other reports from Ghana and other African countries [15, 28]. Two reasons could be ascribed to the high seropositivity detected: 1) high exposure to SARS-CoV-2, or 2) high vaccine efficacy. It is impossible to assess the contribution of true viral exposure to the seropositivity rates observed in this study. This is mainly because of the high number of asymptomatic individuals in the population which makes it difficult to associate symptoms with infection in a cross-sectional community-based survey like we conducted. We were able to show, however, that vaccination is a significant contributor to the seropositivity rates detected. Since the study was conducted at the time when vaccination against SARS-CoV-2 had begun in Ghana, it is encouraging to observe such high numbers of individuals with antibodies, which is a proxy for vaccine efficacy (Table 2). It has also been suggested that there may have been stimulation of the innate response providing partial protective immunity to SARS-CoV-2 acquired from Bacille Calmette-Guerin (BCG) vaccinations African populations [24]. BCG vaccinations have been identified as potent immunomodulators which have been used for treatment and have exhibited protective effect against several respiratory infections [24, 29, 30].

On average, 30-40% of the population mentioned that they adhered to at least one of the established WHO COVID-19 infection prevention guidelines. It is important to note that none of the participants had knowledge of their infection status prior to our visit to their households. As such, they would not be expected to be practicing self-isolation, for example, when they had not been confirmed positive for the virus. All the same for other

general preventative measures such as frequent handwashing, social distancing and avoiding non-essential social contact, it was disappointing to observe a significant majority responding that they did not practice such protocols. It was however encouraging to note that most people who tested positive for the virus wore face masks. For a population that is largely asymptomatic for the disease, wearing of face masks is by far one of the best ways to avoid community spread of the virus.

5. Conclusions

The study demonstrated high seropositivity with low infection positivity to SARS-CoV-2 in a major urban setting in Ghana, as part of studies to understand the population dynamics of the virus in the country. Many people that are positive for the virus are asymptomatic with few febrile symptoms. Combined with low adherence to the COVID-19 prevention protocols, community spread is likely to be more extensive than expected. We have established the importance of continual vaccination efforts for all age groups in reducing viral transmission.

Supplementary Material: The following supporting information can be found as table S1: Summary of socio-demographic information collected.

Authors' contributions: IOD, JA, BA, KAK, SKM and MO conceptualized and designed the study. ESL, MO, YA conducted the survey. ESL, NEA, MO performed laboratory analyses of the samples. ESL, JA, EFG analyzed the data and drafted the manuscript. All authors reviewed, edited, and approved the final manuscript.

Funding: The Bill and Melinda Gates Foundation provided funding for field work and purchase of reagents and consumable for laboratory work.

The World Health Organization provided additional funding for field work, purchase of consumables and also provided the WANTAI ELISA assay kits for the serological analysis

The African Academy of Sciences provided additional funding for field work and the procurement of reagents and consumables

Vincent Munster (VM) is supported by the division of Intramural research of the NIAID.

Institutional Review Board Statement: Procedures in this study conform with the Ghana Public Health Act, 2012 (Act 851) and the Data Protection Act, 2012. Ethical approval (NMIMR-IRB CPN 075/19-20) was obtained from the Institutional Review Board (IRB) of the Noguchi Memorial Institute for Medical Research (NMIMR), University of Ghana.

Informed Consent Statement: Informed consent was obtained from all subjects involved in the study and/or their legal guardians (when subject is below 18 years). All consenting participants agreed to future use of their samples.

Data Availability statement: All data generated or analyzed during this study are included in this published article and its supplementary information files.

Acknowledgements: We acknowledge the technical support of the research staff of Department of Parasitology, Millicent Oye Kyei, Daniel A. Odumang, Grace O. Gyamfi, Millicent S. Afatodzie, Christopher Dorcoo, Joseph Quartey, Sampson Otoo, Jeffery Gabriel Sumboh, Dickson Osabutey, Rahmat bint Yusif Ismail, Edward Dumashie, Isaac Quaye, Prince Horlortu, Juliana Tetteh and Essabella Scott for their involvement in field sampling. We also acknowledge the technical support of Felix Ansah at the West African Centre for Cell Biology of Infectious Pathogens (WACCBIP) and

Preprints (www.preprints.org) | NOT PEER-REVIEWED | Posted: 26 September 2022 doi:10.20944/preprints202209.0390.v1

providing us access to their Quantstudio Real-time PCR equipment. Appreciation goes to Dr. Robert J. Fischer (NIAID-NIH) for technical support. Finally, we acknowledge the support of Nana Yaa Asiedua Appiah, Elizabeth Obeng Aboagye, Benedicta Kwablah Ayertey all of the Infectious Disease Epidemiology Laboratory.

Competing interests: The authors declare that they have no competing interests.

References

1. World Health Organization. WHO Coronavirus Disease (COVID-19) Dashboard With Vaccination Data | WHO Coronavirus (COVID-19) Dashboard With Vaccination Data. World Health Organization. 2021;:1–5. https://covid19.who.int/. Accessed 5 May 2022.
2. Hasell J, Ortiz-Ospina E, Ritchie H, Roser M. "Coronavirus Pandemic (COVID-19)". Our World in Data. https://ourworldindata.org/coronavirus. 2022.
3. Ioannidis JPA. Global perspective of COVID-19 epidemiology for a full-cycle pandemic. European Journal of Clinical Investigation. 2020;50.
4. Zhou P, Yang X Lou, Wang XG, Hu B, Zhang L, Zhang W, et al. A pneumonia outbreak associated with a new coronavirus of probable bat origin. Nature. 2020;579:270–3.
5. Bonney EY, Lamptey H, Puplampu P, Kyei GB. COVID-19 pandemic: Ten research questions Africa must answer for itself. Ghana Med J. 2020;54:107–12.
6. Gilbert M, Pullano G, Pinotti F, Valdano E, Poletto C, Boëlle PY, et al. Preparedness and vulnerability of African countries against importations of COVID-19: a modelling study. Lancet (London, England). 2020;395:871.
7. Over two-thirds of Africans exposed to virus which causes COVID-19: WHO study | WHO | Regional Office for Africa. https://www.afro.who.int/news/over-two-thirds-africans-exposed-virus-which-causes-covid-19-who-study. Accessed 5 May 2022.
8. Paleker M, Tembo YA, Davies M-A, Mahomed H, Pienaar D, Madhi SA, et al. Asymptomatic COVID-19 in South Africa – implications for the control of transmission. Public Heal Action. 2021;11:58–60.
9. Kenu E, Odikro MA, Malm KL, Asiedu-Bekoe F, Noora CL, Frimpong JA, et al. Epidemiology of COVID-19 outbreak in Ghana, 2020. Orig Artic www.ghanamedj.org. 2020;54:5–15.
10. Quashie PK, Mutungi JK, Dzabeng F, Oduro-Mensah D, Opurum PC, Tapela K, et al. Trends of severe acute respiratory syndrome coronavirus 2 (SARS-CoV-2) antibody prevalence in selected regions across Ghana. Wellcome Open Res. 2021;6:173.
11. Salyer SJ, Maeda J, Sembuche S, Kebede Y, Tshangela A, Moussif M, et al. The first and second waves of the COVID-19 pandemic in Africa: a cross-sectional study. Lancet. 2021;397:1265–75.
12. Daria S, Islam MR. The SARS-CoV-2 omicron wave is indicating the end of the pandemic phase but the COVID-19 will continue. Journal of Medical Virology. 2022;94:2343–5.
13. Butt AA, Dargham SR, Chemaitelly H, Al Khal A, Tang P, Hasan MR, et al. Severity of Illness in Persons Infected With the SARS-CoV-2 Delta Variant vs Beta Variant in Qatar. JAMA Intern Med. 2022;182:197–205.
14. Med G. Responding to the COVID-19 pandemic in Ghana. J. 2020;54:72–3.
15. Quashie PK, Mutungi JK, Dzabeng F, Oduro-Mensah D, Opurum PC, Tapela K, et al. Trends of SARS-CoV-2 antibody prevalence in selected regions across Ghana. medRxiv. 2021;26:2021.04.25.21256067.
16. World Health Organization (WHO). Seroepidemiological investigation protocol for coronavirus 2019 (COVID-19) infection. 2020;2020 May:https://www.who.int/publications/i/item/WHO-2019-n.
17. Nemeth R. Respondent selection within the household - A modification of the Kish grid 1 Introduction 2 Reasons for sampling households 3 The Kish grid. Meet young Stat. 2002;:51.
18. Coden E, Russo F, Arosio AD, Castelnuovo P, Karligkiotis A, Volpi L. Optimum Naso-oropharyngeal Swab Procedure for COVID-19: Step-by-Step Preparation and Technical Hints. Laryngoscope. 2020;130:2564–7.

Preprints (www.preprints.org) | NOT PEER-REVIEWED | Posted: 26 September 2022 doi:10.20944/preprints202209.0390.v1

19. Monica Cheesbrough. WHO guidelines on drawing blood : best practices in phlebotomy. World Heal Organ. 2010;:1–105.
20. Samples B. 3.7.2 Collection, Processing, and Shipping of Blood Samples.
21. World Health Organization. COVID-19 Weekly Epidemiological Update. World Heal Organ. 2022; June:1–33.
22. Bwire GM. Coronavirus: Why Men are More Vulnerable to Covid-19 Than Women? SN Compr Clin Med. 2020;2:874–6.
23. Owusu M, Sylverken AA, Ankrah ST, El-Duah P, Ayisi-Boateng NK, Yeboah R, et al. Epidemiological profile of SARS-CoV-2 among selected regions in Ghana: A cross-sectional retrospective study. PLoS One. 2020;15 12 December:1–15.
24. Sharma A, Kumar Sharma S, Shi Y, Bucci E, Carafoli E, Melino G, et al. BCG vaccination policy and preventive chloroquine usage: do they have an impact on COVID-19 pandemic? Cell Death Dis. 2020;11.
25. Kumar S, Thambiraja TS, Karuppanan K, Subramaniam G. Omicron and Delta variant of SARS-CoV-2: A comparative computational study of spike protein. J Med Virol. 2022;94:1641–9.
26. van Doremalen N, Schulz JE, Adney DR, Saturday TA, Fischer RJ, Yinda CK, et al. ChAdOx1 nCoV-19 (AZD1222) or nCoV-19-Beta (AZD2816) protect Syrian hamsters against Beta Delta and Omicron variants. Nat Commun 2022 131. 2022;13:1–12.
27. Shah M, Woo HG. Omicron: A Heavily Mutated SARS-CoV-2 Variant Exhibits Stronger Binding to ACE2 and Potently Escapes Approved COVID-19 Therapeutic Antibodies. Front Immunol. 2022;12 January:1–10.
28. Halatoko WA, Konu YR, Gbeasor-Komlanvi FA, Sadio AJ, Tchankoni MK, Komlanvi KS, et al. Prevalence of SARS-CoV-2 among high-risk populations in Lomé (Togo) in 2020. PLoS One. 2020;15 11 November:1–12.
29. Alhunaidi O, Zlotta AR. The use of intravesical BCG in urothelial carcinoma of the bladder. Ecancermedicalscience. 2019;13:1–9.
30. Wardhana, Datau EA, Sultana A, Mandang V V., Jim E. The efficacy of Bacillus Calmette-Guerin vaccinations for the prevention of acute upper respiratory tract infection in the elderly. Acta Med Indones. 2011;43:185–90.
