# Supplementary material for: A mathematical model of COVID-19 with multiple variants of the virus under optimal control in Ghana
Source: PLoS One. 2024 Jul 2;19(7):e0303791. doi: 10.1371/journal.pone.0303791 (PMC11218976; doi:10.1371/journal.pone.0303791)

1. Initial value for data fitting


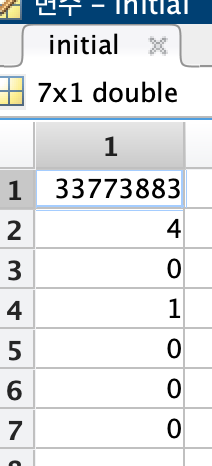
 <- We assume 4 exposed individuals and 1 infected individual on the first day of the outbreak. (I1 case, There is no variant on the first day of onset.)

The day I2 started (Date =='10/20/2021' %start the omicron variants)

1. Control strategy 1 (u1,u2,u3=[a, 0, 0] for a=0.1, 0.5, 0.8)

| Optimal control | Peak I1 | Peak I1 value | Peak I2 | Peak I2 value |
| --- | --- | --- | --- | --- |
| u1=0.1, u2=0, u3=0 | 115 | 2.9275e+05 | 119 | 4.0377e+06 |
| u1=0.5, u2=0, u3=0 | 169 | 6.8763e+03 | 206 | 1.7029e+05 |
| u1=0.8, u2=0, u3=0 | 42 | 31.6601 | 85 | 84.0771 |

1. Control strategy 2 (u1,u2,u3=[0, a, 0] for a=0.1, 0.5, 0.8)

| Optimal control | Peak I1 | Peak I1 value | Peak I2 | Peak I2 value |
| --- | --- | --- | --- | --- |
| u1=0, u2=0.1, u3=0 | 29 | 71.6644 | 35 | 132.6140 |
| u1=0, u2=0.5, u3=0 | 11 | 25.9711 | 12 | 33.6303 |
| u1=0, u2=0.8, u3=0 | 10 | 23.4006 | 11 | 29.5915 |

1. Control strategy 3 (u1,u2,u3=[0, 0, a] for a=0.1, 0.5, 0.8)

| Optimal control | Peak I1 | Peak I1 value | Peak I2 | Peak I2 value |
| --- | --- | --- | --- | --- |
| u1=0, u2=0, u3=0.1 | 114 | 2.1847e+05 | 118 | 2.9303e+06 |
| u1=0, u2=0, u3=0.5 | 140 | 4.5677e+03 | 155 | 5.1806e+04 |
| u1=0, u2=0, u3=0.8 | 99 | 219.5842 | 112 | 1.0128e+03 |

1. Initial value for numerical simulations


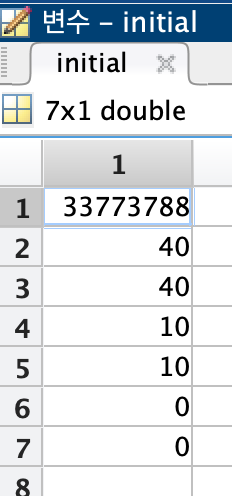

Supplement: S1 File — This material includes the data from Ghana we used and the code written in MATLAB to obtain data fitting results and optimal control results. (ZIP) [file pone.0303791.s003.zip › code_matlab/results/figures/peaks and data.docx]
