# Supplementary figures and images for: A mathematical model of COVID-19 with multiple variants of the virus under optimal control in Ghana
Source: PLoS One. 2024 Jul 2;19(7):e0303791. doi: 10.1371/journal.pone.0303791 (PMC11218976; doi:10.1371/journal.pone.0303791)

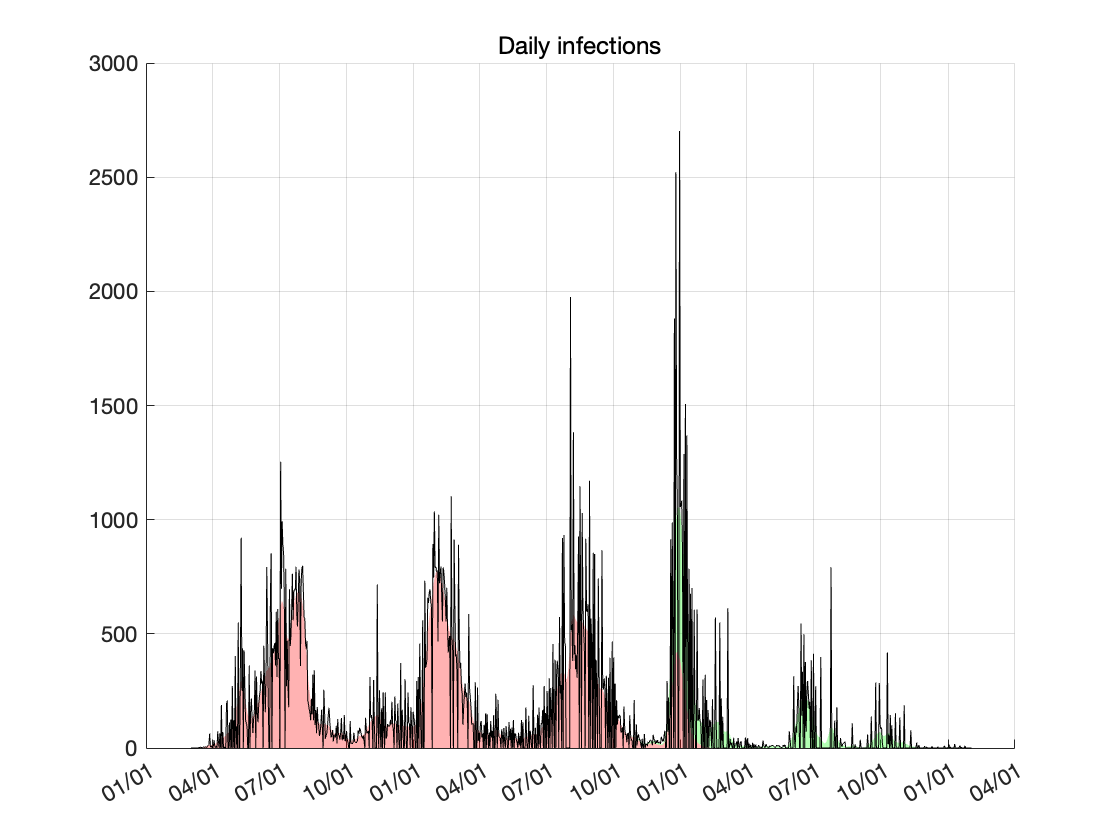

Supplement: S1 File — This material includes the data from Ghana we used and the code written in MATLAB to obtain data fitting results and optimal control results. (ZIP) [file pone.0303791.s003.zip › code_matlab/results/[Fig]Real_data.png]

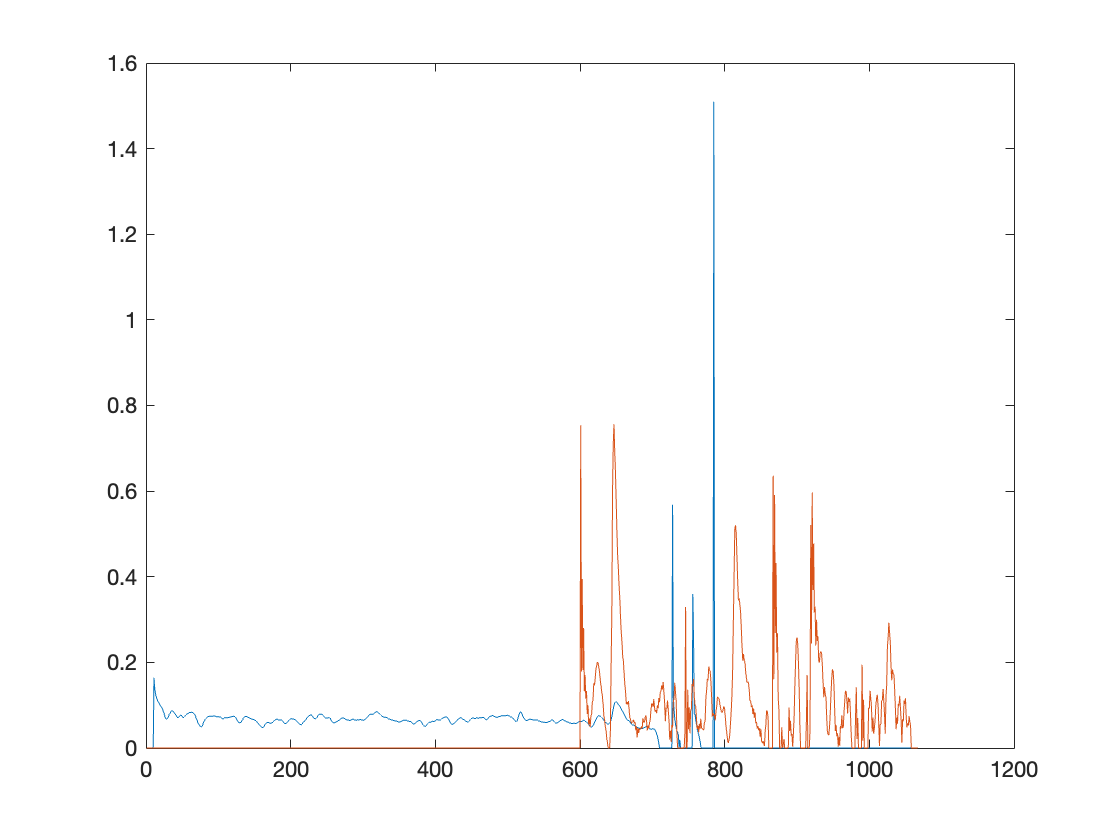

Supplement: S1 File — This material includes the data from Ghana we used and the code written in MATLAB to obtain data fitting results and optimal control results. (ZIP) [file pone.0303791.s003.zip › code_matlab/results/[Fig]Transmission_rates.png]

Daily COVID-19 Confirmed Case Data in Ghana

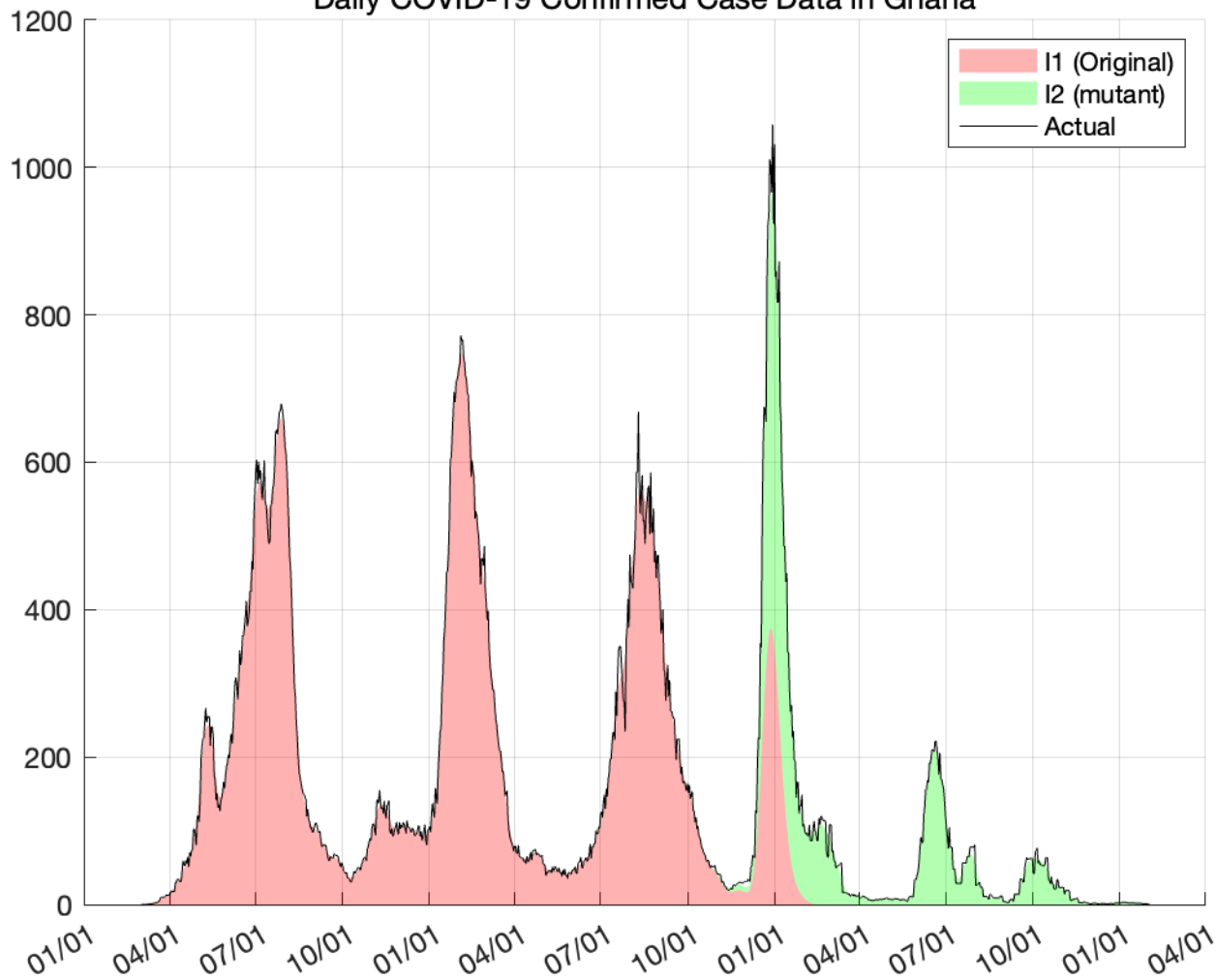

Supplement: S1 File — This material includes the data from Ghana we used and the code written in MATLAB to obtain data fitting results and optimal control results. (ZIP) [file pone.0303791.s003.zip › code_matlab/results/Actual data.pdf]

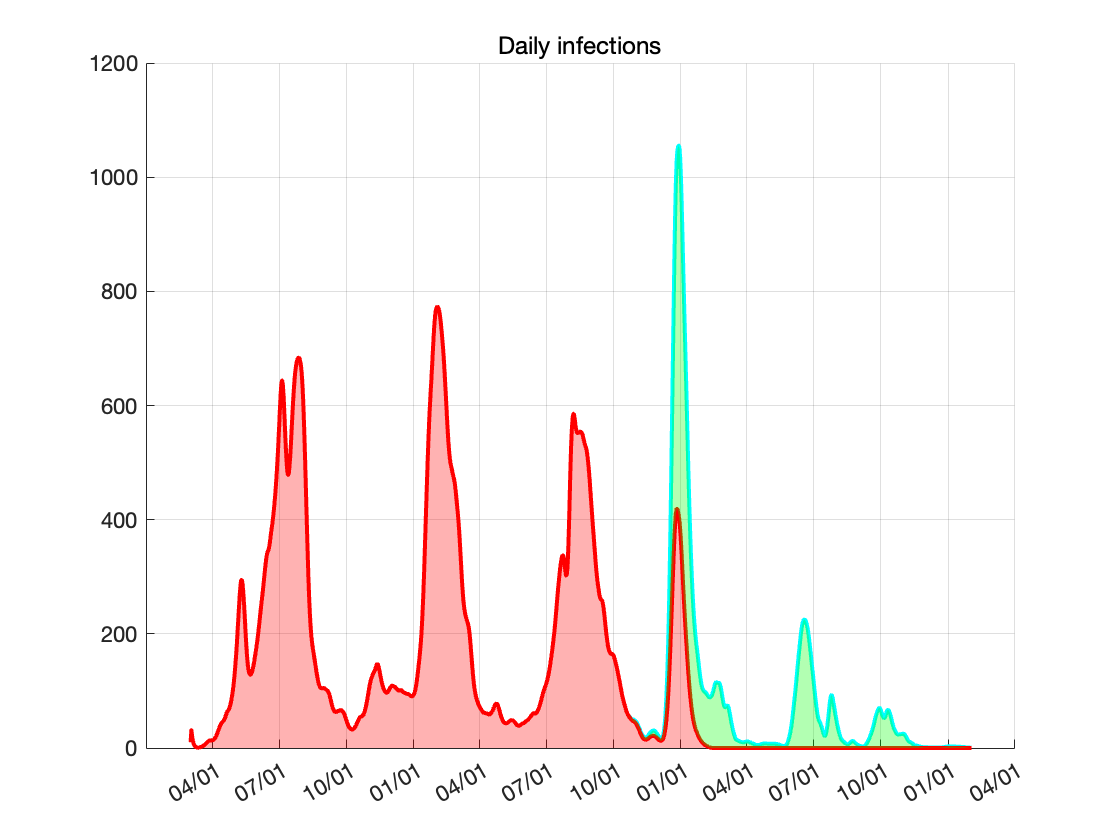

Supplement: S1 File — This material includes the data from Ghana we used and the code written in MATLAB to obtain data fitting results and optimal control results. (ZIP) [file pone.0303791.s003.zip › code_matlab/results/[Fig]Data_fitting.png]

Data fitting of daily COVID-19 confirmed cases in Ghana.

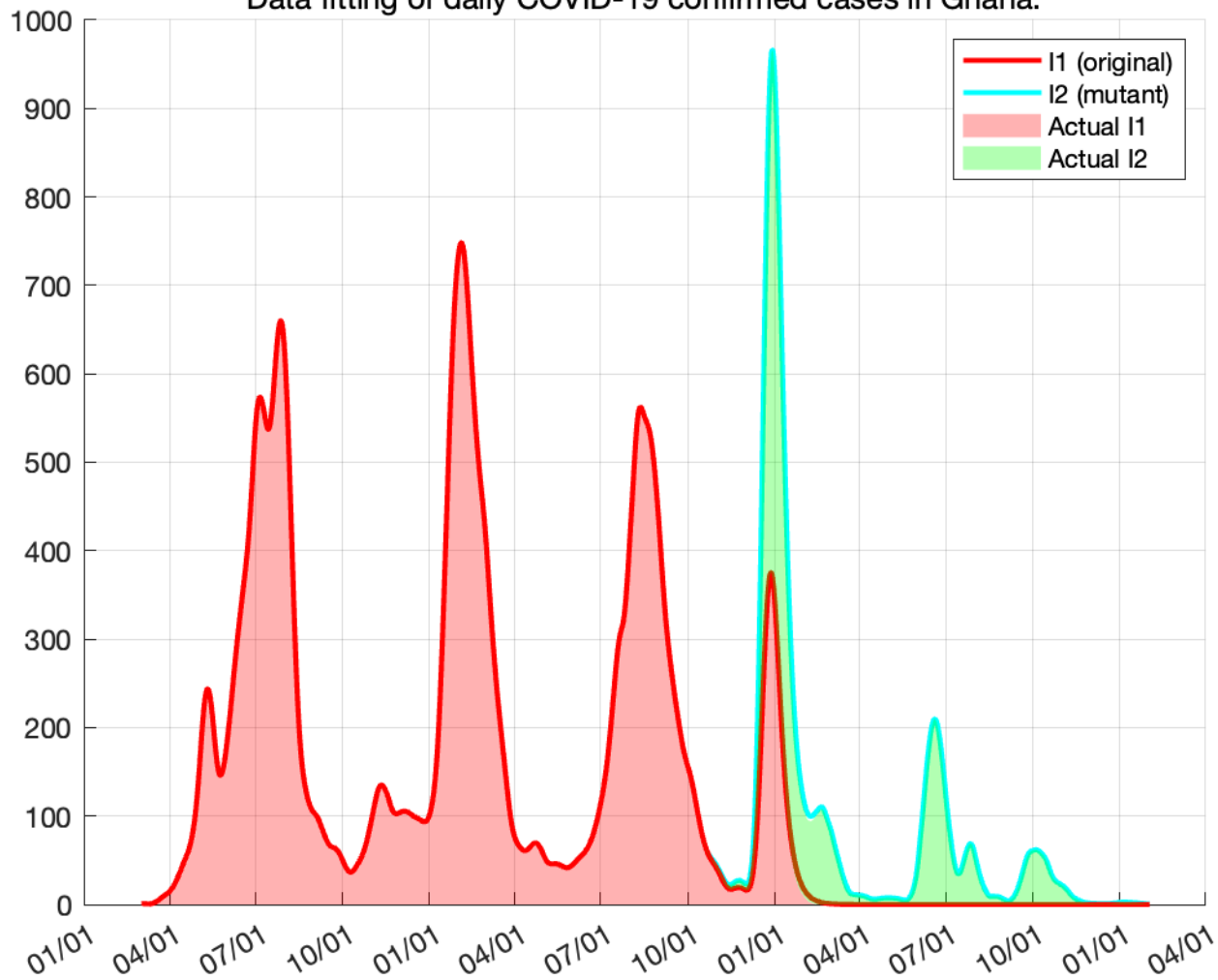

Supplement: S1 File — This material includes the data from Ghana we used and the code written in MATLAB to obtain data fitting results and optimal control results. (ZIP) [file pone.0303791.s003.zip › code_matlab/results/Data fitting.pdf]

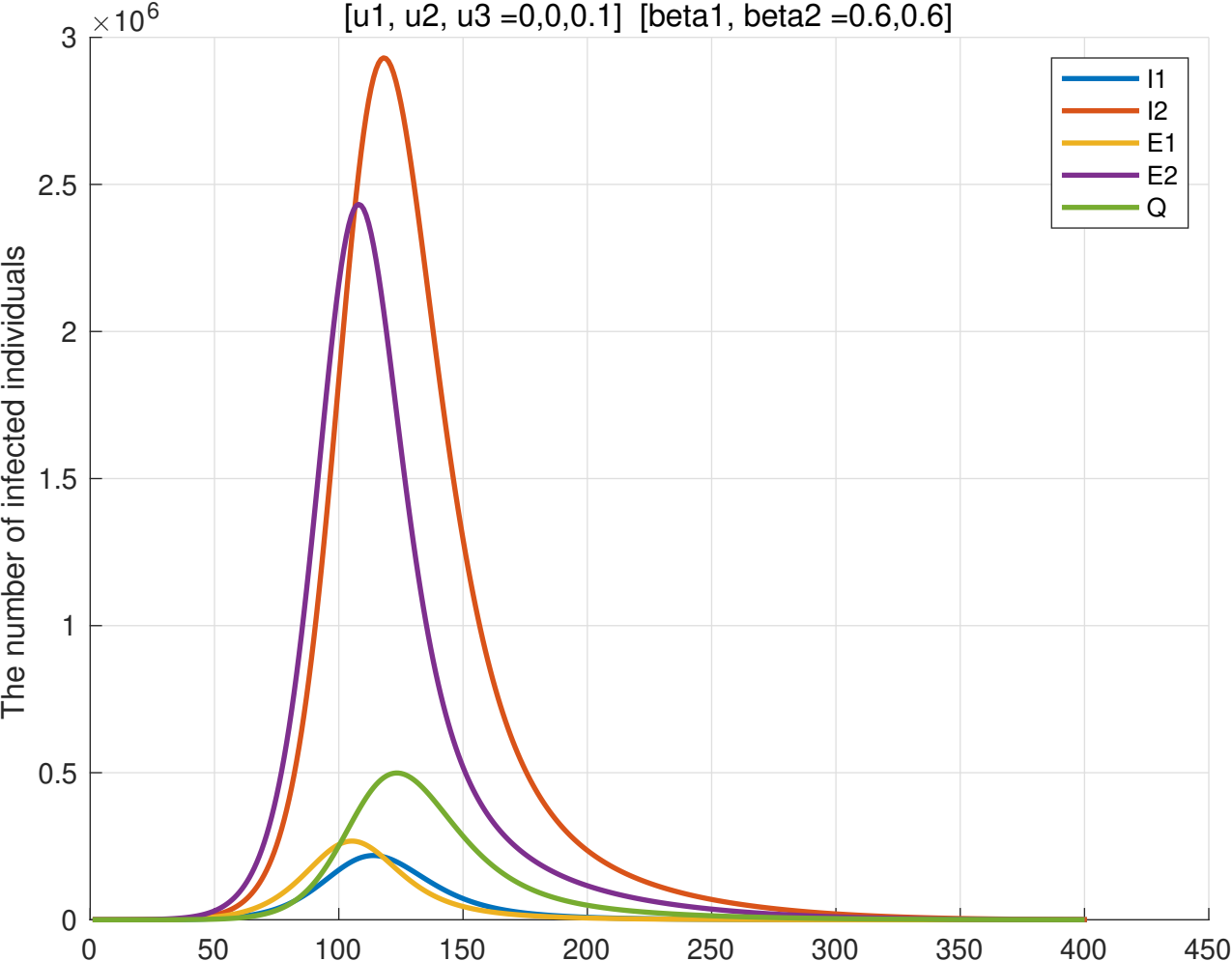

Supplement: S1 File — This material includes the data from Ghana we used and the code written in MATLAB to obtain data fitting results and optimal control results. (ZIP) [file pone.0303791.s003.zip › code_matlab/results/optimal control/[u1, u2, u3 =0,0,0.1] [beta1, beta2 =0.6,0.6].pdf]
