# Supplementary material for: A mathematical model of COVID-19 with multiple variants of the virus under optimal control in Ghana
Source: PLoS One. 2024 Jul 2;19(7):e0303791. doi: 10.1371/journal.pone.0303791 (PMC11218976; doi:10.1371/journal.pone.0303791)
Supplement: Attachment — Submitted filename: Response to Reviewers.pdf [file pone.0303791.s004.pdf]

Answer letter to reviewers

Thank you to the reviewers who took the time to review our research. Thanks to the detailed comments, the quality of our manuscript could be improved. The answers to each reviewer's comments are as follows.

1 Reviewer 1

The results listed in the paper in the form of formulas, figures, and analysis seems true and correct. The paper is well written and it is written in a truly sporty manner. English is generally good, I think it needs to be polished further and some typos need to be revised. Further punctuation marks should be checking through the paper, especially after the equations and at the end of the statements.

1. **Title of paper is not clear. Try to clear meaning of the title.**

Answer Your suggestion is appropriate. We decided to revise the title of our paper to concisely and clearly represent our research topic. The revised title is as follows:
"A mathematical model of COVID-19 with multiple variables of the virus under objective control in Ghana"

2. **The abstract is a little thin and does not quite convey the vibrancy of the findings and the depth of the main conclusions. The authors should please extend this somewhat for a better first impression.**

Answer We supplemented the abstract. Our model had the advantage of being able to control each strain optimally, and the main complement was that we performed various analyses using the multi-strain model. We also explained it in more detail by adding the results of the control strategy and the results that may be interesting.

3. **The manuscript lacks motivation. Author needs to include the motivation of the study.**

Answer Most of the previous studies were conducted under the rapidly changing COVID-19 pandemic. Countries around the world faced an unprecedented emergency for the first time, and implemented various policies to cope with the crisis. However, with sufficient data on currently available diseases, our study motivation was to thoroughly investigate related data from the past in order to prevent the emergence of infectious diseases that threaten humanity, such as COVID-19 pandemic, and to minimize human damage through effective quarantine policies. We presented optimal control strategies for each strain using a multi-strain model and were able to analyze in detail the control strategies that were also implemented. The additions are as follows.

- **In Introduction(Lines 90-100)**: We emphasize that our model is a multi-strain model, comprising two distinct strains. This characteristic enables us to apply optimal control measures comprehensively, allowing for separate analysis of the impact of each strategy on the dynamics of each strain. This facilitates a thorough investigation into the effectiveness of social distancing, vaccinations, and testing-treatment of COVID-19 patients for each specific strain. Such details in our model enhances our ability to tailor and optimize control strategies in response to the dynamics of individual strains. Furthermore, we assess the effectiveness of controls by simulating the combination of two novel control strategies not previously considered. Based on these results, we suggest which strategies may be effective in dealing with newly emerging infectious diseases.

4. **Authors should write keywords in professional way.**

Answer We modified the keywords as follows.

Multi-strain model, COVID-19, social distancing, vaccinations, testing-treatment, optimal control

5. **There is already an abundance of modeling studies on COVID-19, vaccinations, and the months or years to come. However, apart from Ferguson's (now classic) work, Moore and Giordano, very little is said about similar modeling works. This is an issue for three reasons. First, the intended audience for such pieces is made of policy-makers and the general public: they are already facing an abundance of (occasionally conflicting) findings from models. If there is no attempt to contextualize the findings from this piece among others, then we're more likely to be adding noise to a crowded space, instead of providing valuable guidance. Second, several of the modeling assumptions made here may be in line with other pieces (which may provide some strength to the methods) or may be rather unique (which may need more discussion). Finally, as a piece of scientific literature, the contributions should be situated based on what already exists. In sum, the authors should explain how each of their assumptions and modeling choices compares to the literature; how their findings compare to the literature; and hence what is their specific contribution. Related models include, but are not limited to:**

- https://doi.org/10.1016/j.rinp.2021.104285
- https://doi.org/10.1007/s12190-021-01507-y
- https://doi.org/10.1016/j.chaos.2020.110173
- https://doi.org/10.1063/5.0016240
- https://doi.org/10.1016/j.chaos.2020.110049
- arXiv:2005.06286v2
- https://www.researchsquare.com/article/rs-872671/v1
- https://doi.org/10.1140/epjp/s13360-021-01997-6
- https://doi.org/10.1140/epjp/s13360-022-02347-w
- https://doi.org/10.1007/s11071-022-07235-7

Answer Thank you for your detailed feedback. We expect that your comments will improve the quality of our research. The answers are as follows.

(a) We have carefully read all the references you have provided. These studies showed the development of mathematical models for COVID-19 and played a big role in adequately supporting our research motivation. Therefore, we have referred to all of the attached papers in a paragraph starting from the 28th line of Introduction. The difference between our papers, which are revealed based on these papers, is that we have presented optimal control of a multi-strain model that has not been attempted in other models. And all possible combinations of control strategies allowed for more detailed analysis. This was added to the paragraph starting from the 81st line of the manuscript.

(b) We added our model assumptions to the Materials and Methods section (line 138) to emphasize the originality of our model. This clarifies the assumptions used and facilitates comparison with other models. The added assumptions are as follows.

- The SIR model typically assumes a closed population interval. However, as the COVID-19 we are addressing has persisted over an extended period, our model incorporates assumptions concerning births and natural deaths. Newborns are included in the susceptible group, and in each compartment, the population decreases at a rate corresponding to the mortality rate.
- Our model excludes the simultaneous infection with two different strains, operating as a multi-strain model.

- The exposed group (E_i) represents individuals who have been in contact with infected individuals of the i-th strain but have not exhibited symptoms, where $i = 1, 2$. Individuals within this compartment transition to the infected groups (I_i) for each strain based on the transmission rate (β_i).
- The infected groups (I_i) for each strain include both symptomatic and asymptomatic individuals, where $i = 1, 2$. Symptomatic individuals identified in each strain are isolated and transition to the quarantine group at proportions determined by α_1 and α_2. Mildly symptomatic patients and asymptomatic individuals within each strain do not undergo isolation and directly move to the recovery group at a rate determined by γ_i.
- In the infected groups for each strain (I_1 and I_2), we incorporate considerations for the reduction in population due to natural mortality, in addition to the mortality rate attributed to COVID-19.
- Our model assumes the possibility of reinfection as the immunity period ends. Therefore, individuals who have recovered transition back to the susceptible group over time.

6. **Is there any experimental data to validate the mathematical model? The authors at least describe the basic reproduction number R_0 and its impact on covid-19 pandemic. The basic reproduction number R_0 is one of the most crucial quantities in infectious diseases, as R_0 measures how contagious a disease is. In this context the authors include the reference "Mathematical analysis of the global dynamics of a HTLV-I infection model, considering the role of cytotoxic T-lymphocytes, Math. Comput. Simul. 180(2021) 354-378", "Dynamics of an HTLV-I infection model with delayed CTLs immune response, Appl Math Comput 2022"**

Answer Thank you for the valuable feedback. This misunderstanding occurred because we did not mark the real data and the fitted data separately by our mistake. We improved the plot style of the data fitting results and added the phrase fitting results. In addition, we calculated and added the basic reduction number of each fitted strain.

7. **Authors should insert all figures in appropriate places.**

Answer We moved the location of Figure 1 to the model assumption and rearranged the positions of the text and figure. As with the previous problem, we also corrected the Figure 2 in the data preprocessing section. We rearranged the remaining figures according to PLOS ONE's policy.

8. **Conclusion should be written in a more clear way. So try to short it and write in a professional way.**

Answer Based on the reviewer's advice, we have revised the conclusion section. We have retitled the conclusion section as "Discussion and Conclusion" and presented our findings and interpretations of the results in a professional manner.

9. **Analysis is missing in paper so add it.**

Answer Overall, we modified the titles of the manuscript's sections and subsections. We changed the subsections of the Materials and Methods section to A Multi-Strain COVID-19 Model, Model Analysis, and Analysis for Optical Control of the Model.

10. **Authors should improve the English of paper.**

Answer We thoroughly reviewed each sentence of our manuscript. We revised the grammatical expressions and sentences that were difficult to understand. Now it will be easier to read and understand.

11. **Presentation of paper should be improved.**

Answer Overall, the title and composition of the section, the expression of formulas, figures, and tables were improved. For the table showing the results, the numerical expression format was unified and improved to make it easier to recognize. In addition, the format was improved to make the table more clearly represented.

12. **Try to reduce similarity of work.**

Answer We improved the manuscript to reduce the similarity.

13. **References list are not appropriate.**

Answer We further referred to some of the studies and deleted the studies that were not used. Additionally, we checked the references once again and made sure to follow the style of PLOS One.

2 Reviewer 2

I have two major concerns regarding the study.

1. **A very important pathway in the transmission of COVID-19 was the asymptomatic population, this is missing in the model. This makes the model rather unspecific, and in particular may not capture the dynamics of COVID-19 very well.**

Answer Our model is considering asymptomatic cases as well as symptomatic cases. However, unlike some models, our model used the assumption that asymptomatic cases are not assigned separately and asymptomatic cases are moved to the recovered group without quarantine. This is the assumption used in the studies in [1]. We added our model assumptions to the 138th line. We also described in more detail the description of dynamics starting from the 163rd line because it was determined that the description of dynamics was insufficient. Here is the addition of asymptomatic cases in particular.

- However, some of them, either asymptomatic individuals or those with mild symptoms, go straight to compartment R without being tested for COVID-19.

[1] Abioye, A. I., Peter, O. J., Ogunseye, H. A., Oguntolu, F. A., Oshinubi, K., Ibrahim, A. A., & Khan, I. (2021). Mathematical model of COVID-19 in Nigeria with optimal control. Results in Physics, 28, 104598.

2. **The novelty of the study is not clear, most of the work presented already exists in the literature. Perhaps estimation of the contact rates (and hence R_0) is an addition to the existing literature, but this section is not very clear, at the very least fitting results (which it seems have been obtained by fitting several waves) need to be presented in detail.**

Answer The novelty of our study lies in presenting an optimal control model for multiple strains. To clarify this, we added details to Abstract and Introduction. In addition, in order to make our results clearer, we deleted some subsections of the Results unit and reset them to three significant subsections. The results of our study are largely divided into three subsections: model fitting, stability of equilibrium, and control strategies assembly. We will have a clearer view of the results because we only left important subsections.

In particular, the model fitting part you pointed out was misrepresented. This misunderstanding occurred because we did not mark the real data and the fitted data separately by our mistake. We improved the plot style of the data fitting results and added the phrase fitting results. In addition, the basic reproduction number of each fitted strain was added using the equation calculated in the model analysis section.

Further, the language really needs to be fixed, some of the headings and many sentences throughout the study are not grammatically correct.

Answer We thoroughly reviewed each sentence of our manuscript, revising grammatical expressions and sentences that were difficult to understand. Now it will be easier to read and understand. We appreciate the feedback from reviewer and believe that these improvements have enhanced the quality of the paper.
