# Supplementary material for: A mathematical model of COVID-19 with multiple variants of the virus under optimal control in Ghana
Source: PLoS One. 2024 Jul 2;19(7):e0303791. doi: 10.1371/journal.pone.0303791 (PMC11218976; doi:10.1371/journal.pone.0303791)
Supplement: Attachment — Submitted filename: Response to Reviewers.pdf [file pone.0303791.s005.pdf]

Response to Reviewers

We would like to express our sincere gratitude to the reviewers who have kindly reviewed our paper. We have copied your previous review and observations into this document, with responses to each observation highlighted in blue.

Review of "A mathematical model of COVID-19 with multiple variants of the virus under optimal control in Ghana"

I still have several concern regarding the novelty of the work, my previous queries and the author's response are copied below, along with my observations on the authors response.

1. A very important pathway in the transmission of COVID-19 was the asymptomatic population, this is missing in the model. This makes the model rather unspecific, and in particular may not capture the dynamics of COVID-19 very well.

   Answer:

   Our model is considering asymptomatic cases as well as symptomatic cases. However, unlike some models, our model used the assumption that asymptomatic cases are not assigned separately and asymptomatic cases are moved to the recovered group without quarantine. This is the assumption used in the studies in [1]. We added our model assumptions to the 138th line. We also described in more detail the description of dynamics starting from the 163rd line because it was determined that the description of dynamics was insufficient. Here is the addition of asymptomatic cases in particular. – However, some of them, either asymptomatic individuals or those with mild symptoms, go straight to compartment R without being tested for COVID-19. [1] Abioye, A. I., Peter, O. J., Ogunseye, H. A., Oguntolu, F. A., Oshinubi, K., Ibrahim, A. A., & Khan, I. (2021). Mathematical model of COVID-19 in Nigeria with optimal control. Results in Physics, 28, 104598.

Observation

I think asymptomatic carriers are an important characteristic of COVID infections, with estimates in the literature that a high fraction of infected are asymptomatic. At the very least the computational part should include this compartment. I am not sure where in the flow do the authors show susceptibles moving to R, as they seem to imply in their reply.

➔ We assumed that asymptomatic individuals do not undergo COVID-19 testing because they show no symptoms of infection. However, asymptomatic individuals can still transmit the disease through contact with susceptible individuals. Therefore, we included the group of asymptomatic individuals in the group of infected individuals (I_1, I_2), and designed the model such that a subset of asymptomatic individuals among the infected individuals transitions to the recovered group (R) according to the recovery period without isolation measures (hospitalization). To clarify this, and to aid understanding for readers, we improved Figure 1 as follows:

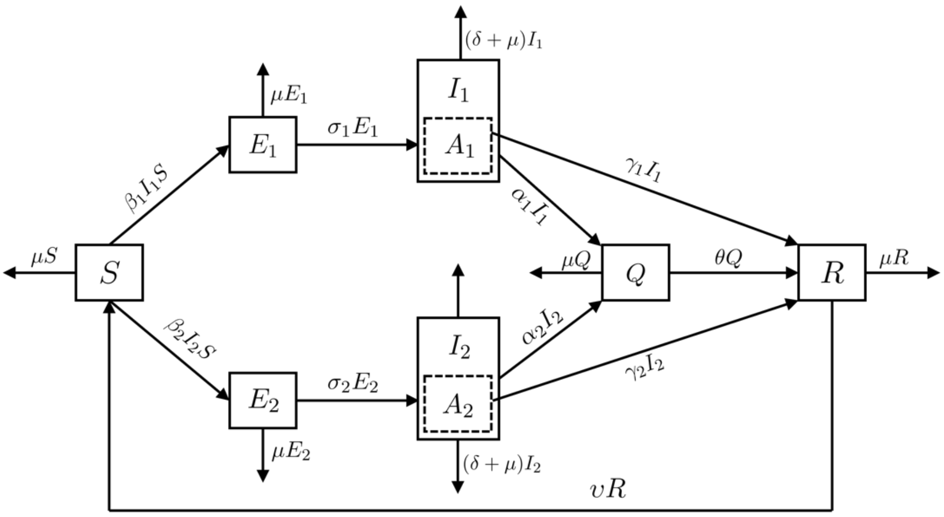

We denoted the asymptomatic group (A_1, A_2) as a subgroup of the infected individual group (I_1, I_2). Additionally, we enhanced the explanation of the parameter gamma and added an introduction to the asymptomatic group in the section introducing compartments of our model.

2. The novelty of the study is not clear, most of the work presented already exists in the literature. Perhaps estimation of the contact rates (and hence R0) is an addition to the existing literature, but this section is not very clear, at the very least fitting results (which it seems have been obtained by fitting several waves) need to be presented in detail.

   Answer:

   The novelty of our study lies in presenting an optimal control model for multiple strains. To clarify this, we added details to Abstract and Introduction. In addition, in order to make our results clearer, we deleted some subsections of the Results unit and reset them to three significant subsections. The results of our study are largely divided into three subsections: model fitting, stability of equilibrium, and control strategies assembly. We will have a clearer view of the results because we only left important subsections. In particular, the model fitting part you pointed out was misrepresented. This misunderstanding occurred because we did not mark the real data and the fitted data separately by our mistake. We improved the plot style of the data fitting results and added the phrase fitting results. In addition, the basic reproduction number of each fitted strain was added using the equation calculated in the model analysis section.

   Observation

   There are many studies in literature where optimal control is used for multiple strains of a disease. Further, I am unclear as to the optimal control results presented, the authors use Pontryagin's principle to calculate the optimal control, but in the subsequent section only constant control at various levels is discussed.

   ➔ As you pointed out, it is possible to adjust the intensity of each optimal strategy over time using our model. Researching optimal strategies over time is crucial for addressing the real-time changes in infectious diseases. However, as emphasized in the introduction section, we focused on comparing the impact of varying the intensity of strategy implementation and discussing which combinations of strategies are more efficient.

Therefore, we needed to fix the levels of each strategy to compare their effectiveness. In this study, we discussed the effectiveness of strategies by measuring factors such as the number of infections occurring at the peak or the duration to reach the peak. Such discussions will be helpful in prioritizing policies and determining the appropriate intensity for implementation when facing new infectious diseases in the future. However, since the points you raised are significant, we have specified them in the discussion and conclusion sections.

3. Further, the language really needs to be fixed, some of the headings and many sentences throughout the study are not grammatically correct.

Answer:

We thoroughly reviewed each sentence of our manuscript, revising grammatical expressions and sentences that were difficult to understand. Now it will be easier to read and understand. We appreciate the feedback from reviewer and believe that these improvements have enhanced the quality of the paper. 4

Observation

The language has been improved, however there are sentences such as "This study suggests a mathematical model…" which are grammatically incorrect, this in itself would not be a major concern as the paper is much more readable now, but for the points earlier.

➔ The sentence you pointed out has been revised as follows.
➔ This study suggests a mathematical model… -> In this paper, we suggest a mathematical model…

To their credit, the authors have done many of the calculations usually performed in such studies. However, how this work adds to the existing

literature on epidemic models in general OR COVID models in particular remains very unclear.

I would strongly recommend that the authors make this a basis for considering a more specific model for COVID OR do a more nuanced data fitting to advance our understanding of COVID dynamics.

- ➔ As an answer to your second observation, we focused on measuring the efficiency of each strategy in the multi-strain model; thus, highlighting those points, we specified how our paper progressed at the end of the introductory section. The following are the added paragraphs.

- ➔ "The paper is organized as follows. In Materials and methods section, we introduce a multi-strain model for COVID-19 constructed from a system of ordinary differential equations. We calculate the model's basic reproduction number and demonstrate the stability of the solution. For the optimal control problems, we show the uniqueness and existence of the solution. In result section, we estimate the transmission rates for each strain using actual data from Ghana using the least squares fitting. We employ these transmission rates to compute the basic reproduction number for each strain. Next, we compare the efficiency of the strategies using an optimal model. We discuss the efficiency of the strategies by measuring the number of infections occurring at the peak and the duration of reaching the peak in various situations. Throughout this process, we conducted a more nuanced data fitting to advance our understanding of COVID-19 dynamics. We look forward to using this study as a basis for considering more specific models for COVID-19."
